# Allosteric Regulation of the Hsp90 Dynamics and Stability by Client Recruiter Cochaperones: Protein Structure Network Modeling

**DOI:** 10.1371/journal.pone.0086547

**Published:** 2014-01-20

**Authors:** Kristin Blacklock, Gennady M. Verkhivker

**Affiliations:** 1 School of Computational Sciences and Crean School of Health and Life Sciences, Schmid College of Science and Technology, Chapman University, Orange, California, United States of America; 2 Department of Pharmacology, University of California San Diego, La Jolla, California, United States of America; Universite de Sherbrooke, Canada

## Abstract

The fundamental role of the Hsp90 chaperone in supporting functional activity of diverse protein clients is anchored by specific cochaperones. A family of immune sensing client proteins is delivered to the Hsp90 system with the aid of cochaperones Sgt1 and Rar1 that act cooperatively with Hsp90 to form allosterically regulated dynamic complexes. In this work, functional dynamics and protein structure network modeling are combined to dissect molecular mechanisms of Hsp90 regulation by the client recruiter cochaperones. Dynamic signatures of the Hsp90-cochaperone complexes are manifested in differential modulation of the conformational mobility in the Hsp90 lid motif. Consistent with the experiments, we have determined that targeted reorganization of the lid dynamics is a unifying characteristic of the client recruiter cochaperones. Protein network analysis of the essential conformational space of the Hsp90-cochaperone motions has identified structurally stable interaction communities, interfacial hubs and key mediating residues of allosteric communication pathways that act concertedly with the shifts in conformational equilibrium. The results have shown that client recruiter cochaperones can orchestrate global changes in the dynamics and stability of the interaction networks that could enhance the ATPase activity and assist in the client recruitment. The network analysis has recapitulated a broad range of structural and mutagenesis experiments, particularly clarifying the elusive role of Rar1 as a regulator of the Hsp90 interactions and a stability enhancer of the Hsp90-cochaperone complexes. Small-world organization of the interaction networks in the Hsp90 regulatory complexes gives rise to a strong correspondence between highly connected local interfacial hubs, global mediator residues of allosteric interactions and key functional hot spots of the Hsp90 activity. We have found that cochaperone-induced conformational changes in Hsp90 may be determined by specific interaction networks that can inhibit or promote progression of the ATPase cycle and thus control the recruitment of client proteins.

## Introduction

Allosteric regulation and support of diverse protein clients underlie the fundamental role of the molecular chaperone Hsp90 in protein synthesis, refolding and degradation [1]–[6]. Hsp90 is an abundant and highly specialized molecular chaperone that is essential for the integrity of many signaling pathways. The rapidly growing body of structural and functional data has significantly advanced the mechanistic understanding of the Hsp90 chaperone that operates in an ATP-coupled functional cycle associated with stochastic switching between structurally different functional states [7]–[13]. A conserved stretch of residues in the nucleotide-binding N-terminal domain (Hsp90-NTD) comprises a “lid” motif that closes over the nucleotide binding site in the ATP-bound closed dimer, while it is in the open conformation in the nucleotide-free and ADP-bound forms of Hsp90. The middle domain (Hsp90-MD) is involved in ATP hydrolysis and contains critical catalytic residues that complement the nucleotide binding site, whereas the C-terminal domain (Hsp90-CTD) is involved in dimerization. Conformational changes in the lid motif are coupled to the ATPase cycle, whereby upon ATP hydrolysis the lid flips away from the nucleotide site and concomitantly the Hsp90 dimer can adopt an open functional form. The functional linkage of the Hsp90 conformational cycle to ATP binding and hydrolysis is essential for its chaperoning function [7]–[13]. However, the kinetics of large conformational changes in yeast Hsp90 is nucleotide-independent, where the formation of the close dimer is the rate-determining step of the reaction [14], [15]. The diverse regulatory mechanisms of the Hsp90 machinery are enabled by the Hsp90 interactions with an array of cochaperones - protein adaptors that are recruited to assist Hsp90 in modulating the progression of the ATPase cycle and chaperoning of the vast protein clientele [16]–[19]. Central to the role of cochaperones is targeted modulation of the ATPase conformational cycle by turning stochastic conformational fluctuations of Hsp90 into precisely engineered progression of specific conformational states that are tailored to structural requirements of protein clients. The class of client recruiter cochaperones can also contribute to the process of client selection and recognition, often by arresting the Hsp90-ATPase cycle in a particular conformational state in order to support activities of specific clients.

Cell division cycle protein 37 (Cdc37) is a highly specialized cochaperone that in coordination with Hsp90 can facilitate protein folding and maintain stabilization of protein kinase clients during maturation until they attain their full biological activity [20], [21]. Conformational changes associated with the recruitment and loading of kinase clients to the Hsp90-Cdc37 chaperone allow kinases to complete maturation of their functional states, initiate subsequent interactions with the protein substrates and activate signaling cascades (Figure S1). Structural and biochemical experiments have indicated that Cdc37-mediated arrest of the Hsp90-ATPase cycle at the early stage would stabilize an open, nucleotide-free conformation of Hsp90 by preventing lid closure and blocking the association of the Hsp90-NTDs [22]–[25]. The human Cdc37 protein structure can be divided into three domains where the N-terminal domain, Cdc37-NTD (residues 1–147) and the middle domain, Cdc37-MD (residues 148–282) recognize protein kinase clients and Hsp90, while the C-terminal domain, Cdc37-CTD (residues 283–378) is primarily involved in Cdc37 dimerization (Figure S2). The middle domain is the most stable region of Cdc37 and contains the Hsp90 recognition site [22]. The crystal structure of the human Cdc37 construct (residues 148–348) in the complex with the yeast Hsp90-NTD [23] has revealed a Cdc37 dimer bound to the “lid” segment of the Hsp90-NTD and intruding into the Hsp90 nucleotide binding pocket (Figure S2A). These interactions formed between the middle domain of Cdc37 and the Hsp90-NTD can inhibit the ATPase activity of Hsp90 by preventing dimerization and disrupting the Hsp90 ATPase cycle [23], [24]. A NMR study of the complex between the middle domain of human Cdc37 (Cdc37-MD, residues 148–276) and human Hsp90-NTD (Figure S2B) has produced a monomeric structure of Cdc37 forming a compact hydrophobic interface with the Hsp90-NTD [25].

**Figure 1:**
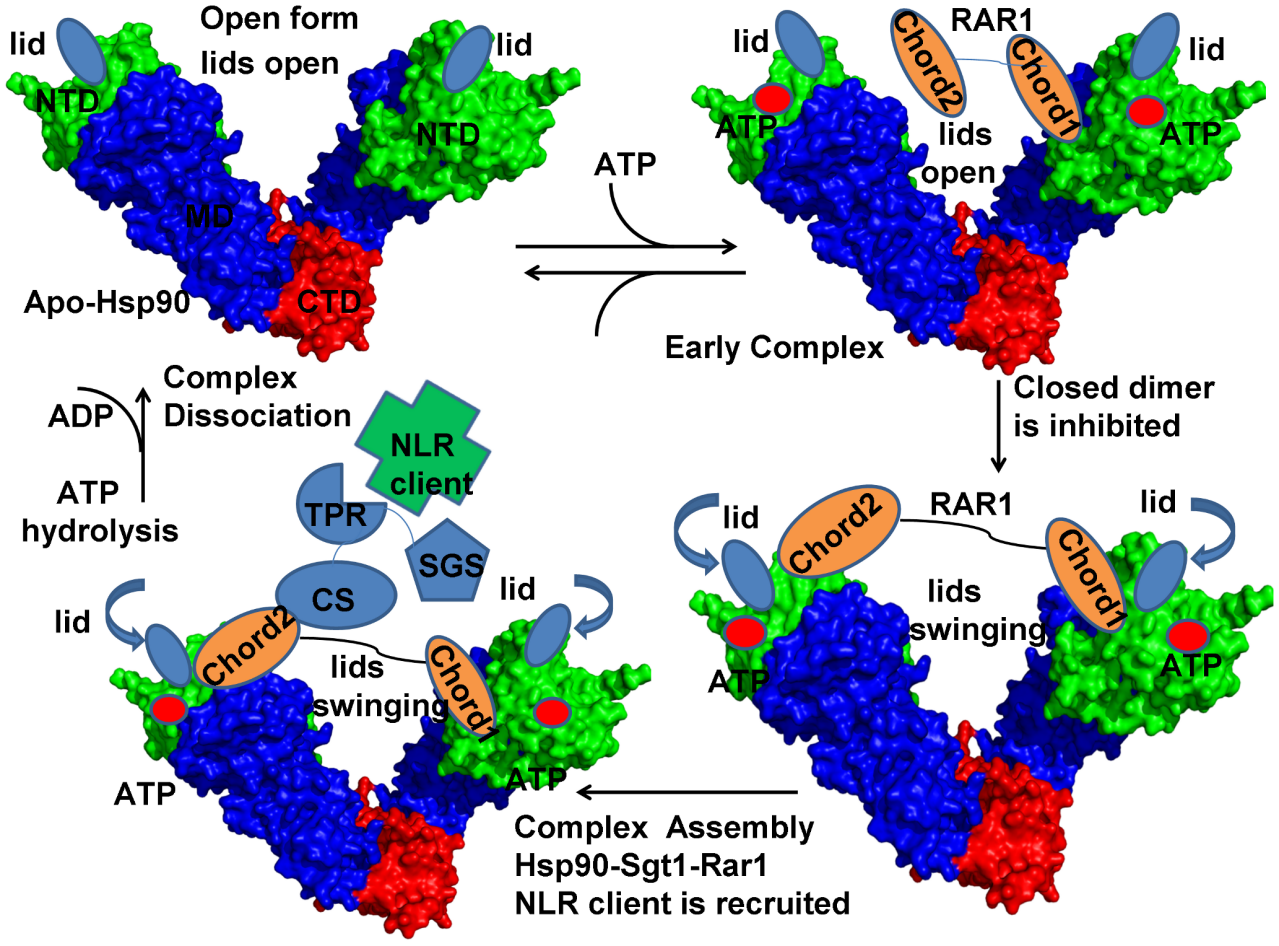
A Model of the Hsp90-ATPase Cycle: The Functional Role of the Cochaperones Sgt1 and Rar1. In this model, ATP binding to the Hsp90-NTD of apo-Hsp90 in the open form can induce a fast dynamic exchange between a nucleotide-free Hsp90 and an ATP-bound state in which the ATP lids and Hsp90-NTDs are still open. Binding of Rar1-CHORD1 to the Hsp90-NTD intersects the normal progression of the ATPase cycle by preventing the lid enclosure of ATP and inhibiting the formation of the closed Hsp90 dimer. This interaction supports the binding of Rar1-CHORD2 to the other Hsp90-NTD in the other protomer. Upon binding of both Rar1-CHORD domains, SGT1 is recruited to interact with the Hsp90-NTD and Rar1-CHORD2 domain. Cochaperone-mediated arrest of the Hsp90-ATPase conformational cycle in the open form promotes the assembly of the ternary Hsp90-Sgt1-Rar1 complex and recruitment of the NLR clients. In the ternary complex, the fluctuations of the lid segment may allow the catalytic Arg residue from the Hsp90-MD to reach the nucleotide binding site and induce ATP hydrolysis. The Rar1-stimulated hydrolysis of ATP would lead to dissociation of RAR1, SGT1, and NLR client from the Hsp90 dimer. After ATP is hydrolyzed, the Hsp90-NTDs domains dissociate and ADP is released returning Hsp90 to the nucleotide-free open state. The Hsp90 structure is shown in a surface representation with an annotation of structural elements. The Hsp90-NTD is shown in green; the Hsp90-MD is depicted in blue and the Hsp90-CTD is presented in red.

Recent studies in plants and mammals have revealed that cochaperones Sgt1 (suppressor of G2 allele of SKP1) and Rar1 (required for MLA12 resistance) are cooperatively integrated into the Hsp90 system for stabilizing NLR (nucleotide-binding domain and leucine-rich repeat containing) proteins, a family of conserved immune sensors that recognize pathogens (Figure 1) [26]–[32]. To defend against foreign pathogens, plants and animals employ these immune sensor proteins which recognize extracellular molecules and initiate immune response. Sgt1 is required for innate immunity and consists of three distinct domains, TPR (tetratricopeptide repeats), CS (CHORD-containing protein and Sgt1) and SGS (Sgt1 specific domain) [33]. NMR studies have demonstrated that human Sgt1 binds the Hsp90-NTD through the Sgt1-CS domain, while the TPR domain is not involved in direct interactions with Hsp90 [34]. The CS domain of Sgt1 (Sgt1-CS) interacts with the Hsp90-NTD and is structurally similar to the p23 (mammals)/Sba1 (yeast) cochaperone, yet the binding sites on the Hsp90-NTD are entirely different [35], [36]. While p23/Sba1 interacts with the closed lid form and stabilizes the ATP-bound conformation of the chaperone, Sgt1 binds to the open lid conformation and has no inherent Hsp90-ATPase regulatory activity [34]–[36]. Rar1 contains two similar cysteine and histidine-rich domains (CHORD1 and CHORD2) that can interact with Hsp90-NTD (Figure 1). NMR-based mapping and mutational analyses of the Sgt1 binding interfaces in plants have confirmed that the Sgt1-CS domain is required for the Hsp90 binding and that Rar1-CHORD2 and Hsp90-NTD interact with the opposite sides of the Sgt1-CS domain [36], [37]. Functionally, Hsp90 and Sgt1 interact with the cochaperone Rar1 which acts as a core modulator in plant immunity. While Sgt1-CS and Rar1-CHORD domains can independently interact with Hsp90, structural and biochemical studies have demonstrated that the Rar1-CHORD2 domain is essential to the formation of the functional complex [38], [39].

Recent crystallographic studies of the core Hsp90–Sgt1 complex [38] and ternary Hsp90-Sgt1-Rar1 complex [40] have provided the first detailed outlook of the architecture of the regulatory complexes, suggesting possible recruitment mechanisms of NLR client proteins, whose role in immune defense is shared in both plants and animals. The crystal structure of the HSP90-Sgt1-Rar1 complex has revealed a heterohexamer in a ring configuration with two copies of each component, in which Rar1-CHORD2 interacts with the Hsp90-NTD opposite to its Sgt1-interacting region [40]. In addition to the interaction between Rar1-CHORD2 and Sgt1, Rar1-CHORD1 binding to the Hsp90-NTD also contributes to the assembly of the Hsp90-Sgt1-Rar1 regulatory complex. According to the structural insights, the dynamically controlled ternary complex is implicated in the activation mechanism of NLRs clients [38]–[40], in which the Rar1-Sgt1 interactions could be critical for disease resistance and the Rar1-Hsp90 interactions may facilitate decomposition of ATP and boost the efficiency of immune sensor control. Collectively, structural and functional studies have suggested a mechanistic model of the NLR recruitment and maturation by the Hsp90-Sgt1-Rar1 complex (Figure 1) [38]–[40]. In this model, ATP binding to the Hsp90-NTD of apo-Hsp90 in the open form can induce a fast dynamic exchange between a nucleotide-free Hsp90 and an ATP-bound state in which the lid segments and the Hsp90-NTDs are in the open position. In the proposed mechanistic picture Rar1 is the key component of the regulatory assembly that can intersect the normal progression of the ATPase cycle at the early stage of the cycle and inhibit the formation of the closed Hsp90 dimer, while accelerating the ATPase activity (Figure 1). Hence, cochaperone-mediated arrest of the Hsp90-ATPase conformational cycle can promote the assembly of the ternary Hsp90-Sgt1-Rar1 complex and recruitment of the NLR clients. Although it is established that the formation of the regulatory Hsp90 complexes with Sgt1 and Rar1 enable targeted modulation of the ATPase conformational cycle, molecular and energetic determinants of allosteric regulation have remained frustratingly elusive. Among important questions that are currently under active investigation are (a) how do Rar1 and Sgt1 cooperate at the molecular level to mechanistically regulate Hsp90? (b) how can Rar1 enhance the ATPase activity of Hsp90? (c) is there a feasible unified mechanism that can explain the maturation process of NLR proteins by the Hsp90-Sgt1-Rar1 complex?

Molecular understanding of the regulatory mechanisms critically depends on high-resolution structures of recognition-competent client states in regulatory complexes with the Hsp90-Sgt1-Rar1 chaperone system. However, the dynamic nature of these molecular assemblies hinders the molecular details that underlie cochaperone-specific modulation of the ATPase activity. Compounded by marginal stability of the regulatory complexes, structural and thermodynamic characterizations of the Hsp90 interactions remain technically challenging, which is evident from a relatively small number of high-resolution structures of the Hsp90-cochaperone complexes. Consequently, computational modeling of transient Hsp90-cochaperone interactions may complement structure-functional studies and provide molecular insights into mechanistic aspects of allosteric regulation of Hsp90. The transient nature and cooperativity of the Hsp90-cochaperone interactions necessitates a multi-scale modeling strategy that combines all-atom and coarse-grained representations of the biological system. The key to understanding dynamics and stability of the regulatory complexes is (a) to provide a quantitative characterization of the dynamics and stability of the Hsp90-cochaperone complexes; and (b) to establish a linkage between cochaperone-induced global conformational changes in Hsp90 and specific interaction networks that can inhibit or promote progression of the ATPase cycle and thus control the recruitment of client proteins.

Although principal modes of protein motions can be extracted from all-atom molecular dynamics (MD) simulations, coarse-grained approaches and elastic network models (ENM) such as Gaussian network model (GNM) [41]–[43] combined with the normal mode analysis [44], [45] can efficiently probe functional movements by reducing protein structure representation to a network of uniformly connected nodes where the native interactions in the equilibrium structure determine conformational dynamics of the system [46]–[48]. Computational studies have employed dynamic approaches to model collective motions and allosteric interactions in the Hsp90 crystal structures revealing conserved functional motifs that act collectively as central regulators of the chaperone dynamics and activity [49]–[55]. All-atom simulations of the Hsp90 crystal structures from different species have detected two inter-domain hinge sites regulating allosteric interactions of the chaperone [56]. Force-distribution analysis based on atomistic simulations has identified an internal signaling pathway connecting the nucleotide binding site in the HtpG via a dynamic hinge with the distantly located client binding region in the middle domain [57]. We have also recently shown that functional dynamics and allosteric interactions of Hsp90 can be selectively modulated by p23 and Aha1 cochaperones via specific targeting of the regulatory hinge regions that could restrict collective motions and stabilize specific chaperone conformations. [58]. Graph-based and network theoretical approaches [59], [60] can further reduce complexity of protein architectures to one-dimensional maps comprised of nodes (residues) connected by edges (inter-residue interactions). This description can yield a convenient characterization of the protein topology and allow for network-based analysis of protein structure. These methods have shown that protein structure graphs are neither regular locally connected graphs, nor they are random locally disconnected graphs that have many long-range edges. It has been recognized that protein topologies and the interaction connectivity could often produce distinct small world networks, which combine the high local connectivity of residue nodes with a smaller number of long-range contacts [59], [60]. Small-world allosteric networks and long-range protein communication can be determined by specific residues playing critical roles in the transmission of functional signals [61], [62]. Network-based analyses have been also used in predicting allosteric communication pathways [61]–[63], protein-protein interactions [64], [65], catalytic site residues in enzymes [66], [67], protein folding mechanisms [68]–[70], and modeling of protein unfolding pathways [71]. Protein networks can be also described as weighted graphs [72]–[74] and use three common measures of node centrality (degree, closeness, and betweenness) introduced in the context of social networks [75]–[79] to identify shortest paths of inter-residue communication. A combination of MD simulations and the protein structure network analysis using a graph-based representation of the residue interactions can identify functionally important sites and subtle structural changes in the conformational populations of states [80]–[87]. Graph-based protein networks that incorporated dynamic contact maps of cross-correlations with the interaction residue connectivity have successfully described allosteric communications in tRNA–protein complexes [88], cysteinyl tRNA synthetase [89], [90], imidazole glycerol phosphate synthase [91], [92], thrombin [93], and the M2 muscarinic receptor [94].

By combining functional dynamics and protein structure network analyses, the reported study attempted to dissect complex mechanisms of Hsp90 regulation by this unique dual co-chaperone system. Coarse-grained modeling was used to identify global dynamic signatures of the Hsp90-cochaperone complexes and show how client recruiter cochaperones can orchestrate global conformational changes in Hsp90 by modulating local dynamics of the lid motif. The variations in the functional dynamics profiles were compared with the evolution of the protein structure networks to determine cochaperone-mediated specific interactions responsible for modulation of the Hsp90-ATPase cycle. We compare distinctive networking profiles of the Hsp90-Cdc37 and Hsp90-Sgt1-Rar1 complexes to illustrate how targeted modulation of the lid dynamics is coupled with specific interactions to inhibit or promote progression of the ATPase cycle. We show that the regulatory Hsp90-cochaperone complexes have a small-world organization of the interaction network in which a group of highly connected interfacial hubs and global mediator residues of allosteric communications could serve as functional hot spots of the Hsp90-ATPase activity.

## Results and Discussion

### Sgt1 and Rar1 Cochaperones Differentially Modulate Conformational Mobility of the ATP Lid

**Figure 2:**
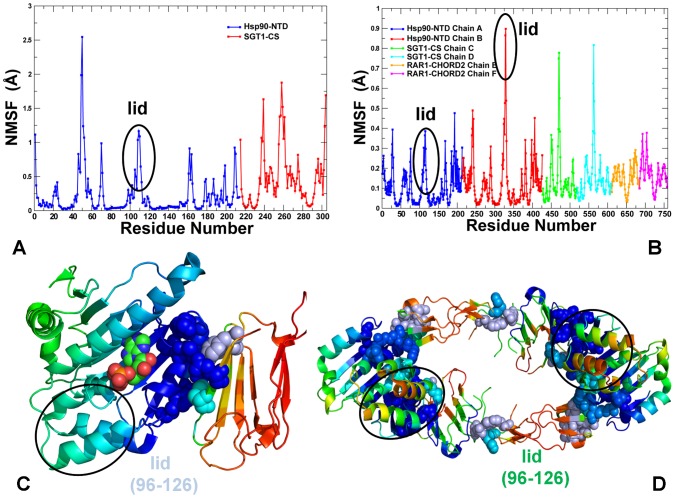
The Residue-Based Fluctuation Profiles of the Hsp90-Sgt1 and Hsp90-Sgt1-Rar1 Complexes. The residue-based NMSF profiles of the Hsp90-Sgt1 (A) and Hsp90-Sgt1-Rar1 complexes (B) were computed by averaging the fluctuations over 20 low frequency modes. In the Hsp90-Sgt1 complex, the NMSF profile of the Hsp90-NTD residues is shown in blue lines and the NMSF values for the Sgt1-CS residues are depicted in red lines. The consecutive residue numbering of the Hsp90-NTD and Sgt1-CS residues is adopted. The original numbering of the Hsp90-NTD residues (residues 4–217) from the crystal structure (PDB ID 2JKI) corresponds to residues 1–213 in panel (A). The crystal structure annotation of the Sgt1-CS domain (residues 151–240) corresponds to residues 215–304 in panel (A). The crystal structure of the HSP90-Sgt1-Rar1 complex (PDB ID 2XCM) is a heterohexamer with two molecules of each domain (B). The NMSF profiles for the Hsp90-NTD domains are shown in blue (molecule 1) and red (molecule 2); the NMSF graphs for the Sgt1-CS domain are in green (molecule 3) and cyan (molecule 4); the NMSF plots for the Rar1-CHORD2 domain are in orange (molecule 5) and magenta (molecule 6). The crystal structure residue numbering was converted to a consecutive numbering. The Hsp90-NTD residues are 1–213 (molecule 1) and 214–426 (molecule 2). The crystal structure numbering for the Sgt1-CS domain (residues 150–241) translates in consecutive residues 427–518 (molecule 3) and 519–610 (molecule 4). The crystal structure numbering for the Rar1-CHORD2 domain (residues 148–221) converted to residues 611–684 and 685–758. Structural distribution of conformational mobility in the essential conformational space of the Hsp90-Sgt1 (C) and Hsp90-Sgt1-Rar1 complexes (D) was obtained by averaging the residue fluctuations along the three lowest frequency modes. A surface-based protein representation is employed. The color gradient from blue to red indicates the decreasing structural rigidity (or increasing conformational mobility) of protein residues. The interfacial residues are shown spheres and colored according to their mobility. The ADP in the Hsp90-Sgt1 complex (C) is shown in atom-based colored spheres. The position and conformational mobility of the lid motif in the Hsp90-Sgt1 and Hsp90-Sgt1-Rar1 complexes are highlighted and pointed to by oval circles surrounding the lid.

In the crystal structure of the binary Hsp90-Sgt1 complex [38], the lid segment of the Hsp90-NTD is ordered in its open conformation with the relative thermal parameters comparable to the structurally rigid core of the Hsp90-NTD domain. However, the Hsp90-NTD binding site for the Sgt1-CS domain does not overlap with the nucleotide binding site and hence, would not directly interfere with the lid conformation (Figure 2). In contrast, the lid motif exhibited significantly higher thermal parameters and was appreciably disordered in the crystal structure of the regulatory ternary Hsp90-Sgt1-Rar1 complex [40]. Based on the crystal structure analysis, it was proposed that the Rar1-CHORD2 domain can function as a regulatory on-off switch of the lid mobility by favoring a flexible lid conformation in the ternary complex [32], [40]. We carried out functional dynamics analysis of the Hsp90-Sgt1 and Hsp90-Sgt1-Rar1 complexes to (a) characterize cochaperone-specific modulation of the lid dynamics, and (b) understand how allosteric interactions with the lid motif may allow cochaperones to engineer global conformational changes and control the ATPase activity. According to our working hypothesis, the thermal fluctuations of the inherently mobile lid could be present in both complexes. Rather than operating an on-off switch, we proposed that Rar1-CHORD2 may interfere in the conformational equilibrium acting as a “relay switch” by selectively reducing or enhancing the relative conformational mobility of the lid motif. Functional dynamics and collective protein motions are largely determined by the native interactions and low frequency normal modes of fluctuations around the equilibrium structure. The crystal structures of the Hsp90-Sgt1 and Hsp90-Sgt1-Rar1 complexes were optimized using the 3Drefine method [95] that is based on an atomic-level energy minimization using a composite physics and knowledge-based force fields. This approach allows for a robust refinement of the global topology and the side-chain interaction networks in the final structures that are relatively insensitive to the energy force field. We compared the dynamics of the Hsp90-Sgt1 and Hsp90-Sgt1-Rar1 complexes by relying on the transferability of the ENM-derived lowest normal modes that could adequately reproduce functionally important motions.

Conformational mobility of the Hsp90-cochaperone complexes was first evaluated by using the normalized mean square residue fluctuations (NMSF) obtained from computation of the 20 low frequency modes (Figure 2). As expected, the aggregate dynamic profiles revealed that the lid region (residues 96–126) could remain fairly mobile in both the Hsp90-Sgt1 (Figure 2A) and Hsp90-Sgt1-Rar1 complexes (Figure 2B). It may be noticed that the relative fluctuations of the lid segment with respect to the structural core in the ternary complex were considerably larger and somewhat asymmetrical, causing larger deviations in the second Hsp90-NTD molecule (Figure 2B). The NMSF profile of the Hsp90-Sgt1-Rar1 complex displayed more distinct characteristic peaks in the lid region which are indicative of the sustained mobility during functional movements. By projecting conformational mobility profiles onto the subspace of the three lowest frequency modes, we could disentangle critical differences in functional movements of the Hsp90-cochaperone complexes. Generally, we observed that the interfacial residues in both binary and ternary complexes (shown in spheres and colored according to their mobility in Figure 2C, D) experienced an appreciable reduction in conformational mobility. Conformational mobility of the lid motif in the Hsp90-Sgt1 complex remained to be present, but was greatly reduced in the essential subspace (Figure 2C). Consistent with the NMR mapping of the Hsp90-NTD-Sgt1-CS binding surface [36], [37], a number of interacting Sgt1-CS residues displayed a significantly decreased mobility including E155, Y157, Q158, K159, F168, and L218 (Figure 2C). Concurrently, we noticed that the non-interacting regions of the Sgt1-CS domain were rather flexible, perhaps reflecting larger movements of the cochaperone domain in the Hsp90-Sgt1 complex. In agreement with structural studies [36]–[40], we observed a considerable stabilization of the Hsp90-NTD residues extending beyond the firmly rigid interfacial residues (E6, F8, T87, K88, H142, D145, and Y148) and reaching to the non-interacting regions (Figure 2C). Structural stability of the lid segment in the Hsp90-Sgt1 complex is not uniformly distributed and a small connecting loop remained to be partly flexible in the complex. Hence, the Sgt1-induced modulation of the lid motions in the Hsp90-Sgt1 complex may attenuate conformational mobility of the lid rather than acting as a binary switch between rigid and flexible forms. This is consistent with the experimental evidence that the interaction between the Hsp90-NTD and SGT1-CS domains could be weakly inhibited by the AMP-PNP analog [38]. In other words, while Sgt1-CS preferentially binds to the open lid conformation in the ADP-bound Hsp90-NTD, the cochaperone may still associate with the closed (or intermediate) lid conformations albeit with lower affinity.

**Figure 3:**
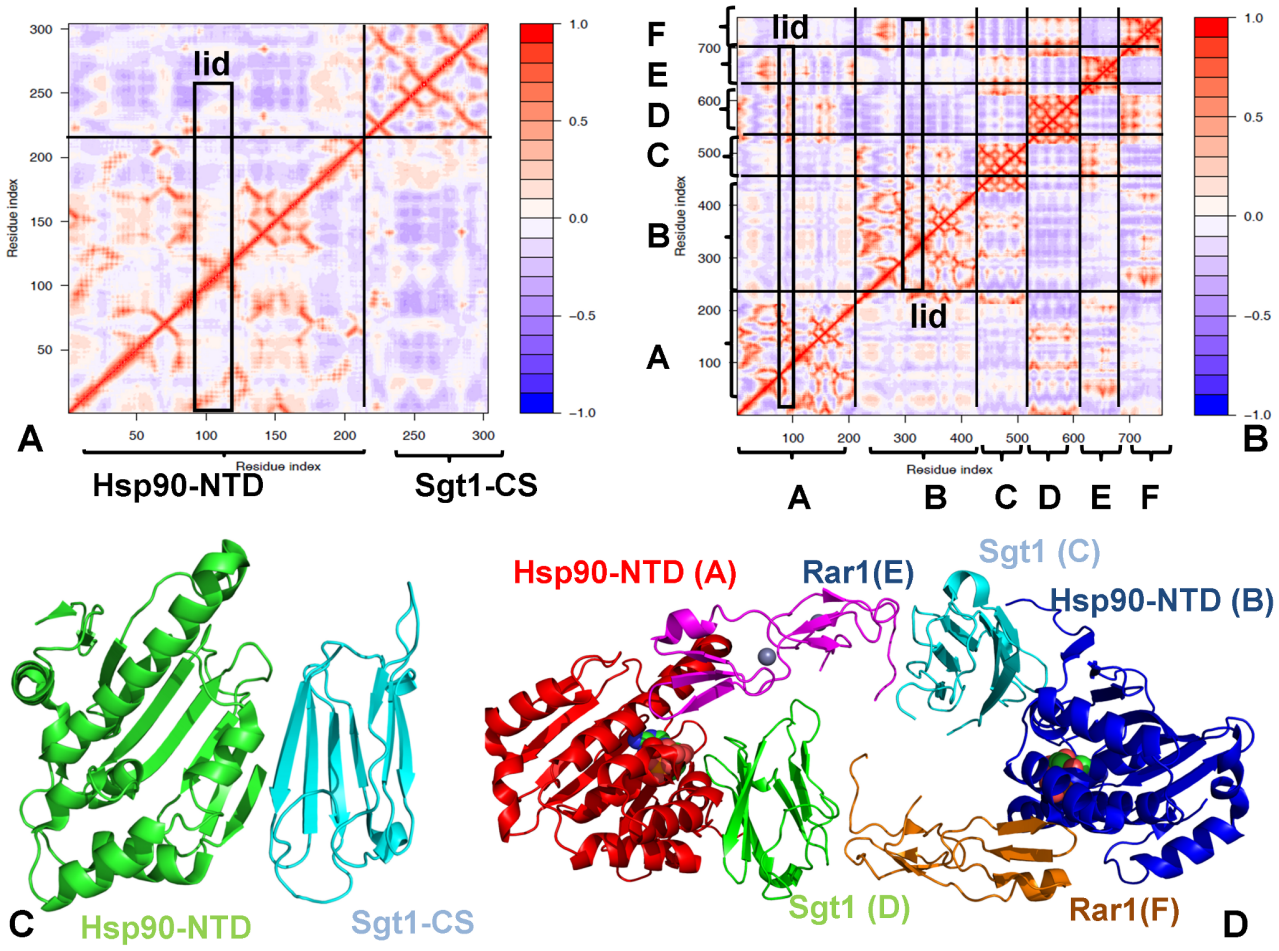
Analysis of the Correlated Motions in the Hsp90-Sgt1 and Hsp90-Sgt1-Rar1 Complexes. The cross-correlation matrices of residue fluctuations in the Hsp90-Sgt1 (A) and in the Hsp90-Sgt1-Rar1 complex (B). The matrix was calculated using the ENM-derived normal modes of the refined structures. The axes denote Cα atoms of the protein residues in sequential order, so that each cell in the plot shows the isotropic correlation of two residues in the protein. Cross-correlations of residue-based fluctuations vary between +1 (fully correlated motion; fluctuation vectors in the same direction, colored in red) and −1 (fully anti-correlated motions; fluctuation vectors in the same direction, colored in blue). The consecutive residue indexing of the Hsp90-cochaperone complexes is adopted and is consistent with the detailed annotation in Figure 2. The residue ranges for the domains in the Hsp90-Sgt1 and Hsp90-Sgt1-Rar1 complexes are mapped onto correlation maps. The position of the lid motif is indicated by a rectangular and the correlation of the lid with the rest of the protein is highlighted. (C) The Hsp90-Sgt1 structure is shown in a ribbon representation I (Hsp90-NTD in green, and Sgt1-CS in red). (D). The Hsp90-Sgt1-Rar1 structure is in ribbons with the Hsp90-NTD domain in red (molecule A) and blue (molecule B). The Sgt1-CS domains are shown in cyan (molecule C) and green (molecule D). The Rar1-CHORD2 domains are depicted in magenta (molecule E) and orange (molecule F).

In a clear contrast, the open conformation of the lid in the Hsp90-Sgt1-Rar1 complex appeared to be as flexible as the most labile loop regions (Figures 2B, D). We also noticed that the lid segment could be easily displaced from the open conformation during low frequency motions, and migrate between alternative states without affecting structurally stable core of the ternary complex (Figure 2D). The greater flexibility of the Sgt1 molecule in the binary complex could be associated with the lack of correlated intermolecular motions (Figures 3A, C). Hence, thermal fluctuations of the lid in the Hsp90-Sgt1 complex may be largely decoupled from the movements of the Sgt1-CS domain. This dynamic signature of the binary complex may be associated with the fact that the binding of Sgt1-CS is not sufficient for modulation of the ATPase activity and Rar1 needs to be recruited to form a fully functional ternary complex [38]–[40]. The increased flexibility of the lid in the ternary complex is consistent with the notion that the lid may fluctuate between an open conformation and an intermediate swinging conformation (Figure 3B,D) [32], [40]. These results support the model of the ATPase cycle (Figure 1), according to which the thermal movements of the lid segment may open up the conformational space for the catalytic Arg residue from the Hsp90-MD to reach the nucleotide binding site and induce ATP hydrolysis. Although the lack of structural information about the full Hsp90 dimer interacting Sgt1 and Rar1 precludes direct modeling of this mechanism, our results point to the key role of Rar1-CHORD2 as an allosteric regulator of the lid dynamics. Rather interestingly, despite a considerable lid flexibility in the ternary complex, the lid movements in each of the Hsp90-NTD could be correlated with the functional displacements of the interacting Sgt1-CS and Rar1-CHORD2 domains (Figure 3D). Hence, in contrast to the binary complex, functional dynamics of the Hsp90-Sgt1-Rar1 assembly may be characterized by correlated motions of the interacting molecules. These findings are consistent with the experiments suggesting that RAR1-CHORD2 facilitates cooperative assembly of the complex and can enhance the ATPase activity while destabilizing the closed lid conformation [38], [40].

### Dynamic Coupling of Structural Rigidity and Flexibility in the Hsp90-Cochaperone Complexes

**Figure 4:**
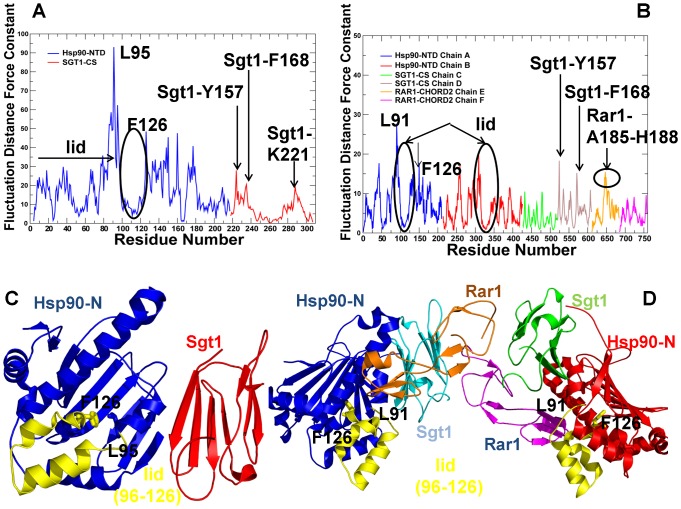
The Force Constant Profiles of the Hsp90-Sgt1 and Hsp90-Sgt1-Rar1 Complexes. The dMD-derived fluctuation distance force constant profiles of the Hsp90-Sgt1 (A) and Hsp90-Sgt1-Rar1 complexes (B) were computed as implemented in [96]. The peaks in the distributions can correspond to the hinge sites. In the Hsp90-Sgt1 complex, the residue-based force constant profile of the Hsp90-NTD residues is shown in blue lines and the profile for the Sgt1-CS residues is in red lines. The force constant graph of the Hsp90-Sgt1-Rar1 complex is consistent with the coloring adopted in Figure 2. The consecutive residue numbering is adopted in both complexes and is consistent with the annotation in Figure 2. The position of the lid motif and the peaks corresponding to functionally important residues are indicated by arrows and annotated. The crystal structures of the Hsp90-Sgt1 (C) and Hsp90-Sgt1-Rar1 complexes (D) are annotated according to the domain coloring adopted in (A) and (B). The lid motif in both structures is highlighted and colored in gold; the anchoring residues with the high force constants are indicated. To streamline the comparison with structural and functional experiments, we annotated functional residues according to their original numbering in the crystal structures.

According to previous studies [49]–[58] structural plasticity and functional adaptation of Hsp90 to the vastly divergent families of interacting cochaperones and client proteins are enabled by modulating the proper balance of structural rigidity and flexibility in the Hsp90 interdomain interfaces. We have recently demonstrated that functionally important regulatory sites of Hsp90 may be strategically positioned at the interdomain regions separating structurally rigid and flexible regions [58]. These residues often correspond to hinge sites around which large protein movements are organized. In this section, we analyzed how cochaperone-mediated modulation of the Hsp90 dynamics could affect the distribution of structural rigid and flexible regions that are crucial for proper functioning of the chaperone. Using the force constant method [96] within the framework of the discrete molecular dynamics formalism [97]–[99] as implemented in [100] we computed the fluctuation distance force constant for each residue in the Hsp90-Sgt1 (Figure 4A) and Hsp90-Sgt1-Rar1 complexes (Figure 4B). The highest sharp peaks in force constant distributions are typically associated with the residues forming boundaries between structurally rigid and flexible regions, and could indicate the interdomain hinge sites [96]. The residue-based force constant profiles of the Hsp90-Sgt1 and Hsp90-Sgt1-Rar1 complexes are characterized by several high value peaks separating structurally rigid and flexible residues (Figure 4). Interestingly, the most notable sharpest peaks that signify an abrupt transition from structurally stable to mobile regions were observed near the lid motif of the Hsp90-NTD. In the Hsp90-Sgt1 complex, the pronounced peak corresponds to the L95 residue that anchors one end of the lid motif and F126 that anchors the opposite end of the lid (Figures 4A, C). Only a few very minor peaks could be spotted in the Sgt1-CS domain, corresponding to a stretch of structurally immobilized residues at the intermolecular Hsp90-Sgt1 interface. Consistent with the ENM-based analysis, the force constant profile of the binary Hsp90-Sgt1 complex similarly indicated the greater mobility of the Sgt1-CS domain.

The residue-based dynamic profiles are based on the consecutive residue numbering for the Hsp90-NTD and Sgt1-CS domains in the complex. For clarity of the comparison with structural and functional experiments, we refer to the important functional residues according to their original numbering in the crystal structures. We observed three distinctive peaks in the Sgt1-CS domain profile corresponding respectively to Y157, F168, and K221 residues (Figure 4A). All these residues are important functional hot spots as the alanine mutations of Y157 and F168 or the charge reversal on K221 could abolish the Hsp90-Sgt1 binding and significantly impair the essential Sgt1 functions in yeast [38]. In the Hsp90-Sgt1-Rar1 complex, the force constant profile similarly revealed the highest peaks corresponding to L91 and F126 from the Hsp90-NTD, thus indicating that the border residues of the lid motif separate structurally rigid core of the ternary complex from the floppy lid residues (Figures 4B, D). The characteristic force constant peaks in the Sgt1-CS domain were preserved between binary and ternary complexes and corresponded to the Y157, F168, and K221 residues (Figure 4B). As a result, these Sgt1-CS residues could form conserved functional sites that are shared in the Hsp90-Sgt1 and Hsp90-Sgt1-Rar1 complexes. These results are consistent with the NMR studies [36], [37], according to which the Sgt1 residues whose resonances were shifted in the Hsp90-Sgt1 complex would not be further perturbed by the addition of the Rar1-CHORD2 domain since the binding mode of the Hsp90-Sgt1 complex is unaffected by RAR1-CHORD2. Another noticeable peak could be seen in the Rar1-CHORD2 domain that identified a stretch of residues A185-H188 as a cohesive structurally stable site during functional movements of the ternary complex (Figure 4B). Indeed, the side-chain of H188 is involved in direct interactions with ADP in the crystal structure, whereas yeast two-hybrid analysis of the Rar1-CHORD2 binding determined that mutations of A185 and H188 severely compromised the interactions and stability of the functional complex [40].

In both complexes, the force constant profile is characterized by a rather steep and narrow well that corresponds to the lid residues and indicative of their mobility in both complexes (Figure 4). Interestingly, the force constant peaks in the Hsp90-NTD correspond to the anchoring residues of the lid motif L95/F126 in the Hsp90-Sgt1 complex and L91/F126 in the ternary complex. The projection of the force constant profiles onto the lowest frequency modes indicated that the lid motif in the Hsp90-Sgt1 complex may maintain a stable open form during functional movements. In contrast, in the Hsp90-Sgt1-Rar1 complex, the lid continues to retain a considerable degree of conformational mobility that may be inferred from low force constant values of lid residues (Figures 4B, D). Hence, two different coarse-grained models revealed a consistent pattern of the lid dynamics. Both the force constant analysis and the ENM-based conformational mobility profile of the Hsp90-cochaperone complexes demonstrated that the enhanced conformational mobility of the lid may be a salient characteristic of the Hsp90-Sgt1-Rar1 complex. These results support our hypothesis that Sgt1-CS and Rar1-CHORD2 may differentially modulate conformational mobility of the lid motif. In the functional ternary complex, the lid motif may freely fluctuate between open and closed forms, likely spanning a range of intermediate conformations and allowing a transient access to the nucleotide binding site in the absence of bound ATP. As a result, the Rar1 binding may destabilize the closed lid form and eliminate the slow step of the formation of the Hsp90 dimer. We propose a model in which cochaperone-mediated regulation of the lid dynamics could be reminiscent of a “rheostat-like” (or “dimmer”) mechanism that adjusts the lid mobility accordingly to engineer precise changes in the ATPase activity. In this mechanism, the Hsp90-cochaperone system may successfully “bypass” stochastically-driven slow conformational changes of the Hsp90 dimer and facilitate ATP hydrolysis. These results provide some support to the recently proposed mechanistic picture [32] in which Rar1-mediated interactions may enhance the ATPase activity by decoupling ATP hydrolysis from the conformational changes in the Hsp90 dimer.

**Figure 5:**
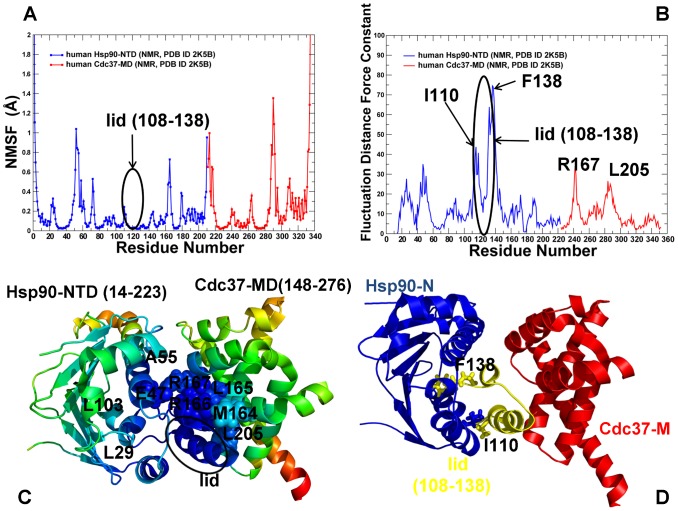
Conformational Mobility Profiling of the Hsp90-Cdc37 Complex. The NMSF profile of the Hsp90-Cdc37 complex (A) was obtained using the NMR structure (PDB ID 2K5B) of the complex between human Cdc37 (Cdc37-MD, original residue numbering 148–276) and human Hsp90-NTD (original residue numbering 14–223) [25]. The residue-based NMSF values were computed by averaging the fluctuations over 20 low frequency modes. The NMSF profile for the Hsp90-NTD residues is shown in blue lines and for the Cdc37-MD residues in red lines. The consecutive residue numbering of the Hsp90-NTD and Cdc37-MD residues is adopted. The original numbering of the Hsp90-NTD (residues 14–223) in the NMR structure corresponds to residues 1–210, and the original numbering of the Cdc37-MD (residues 148–276) corresponds respectively to residues 211–339. (B) The fluctuation distance force constant profile of the Hsp90-Cdc37 complex. The profile is shown in blue lines for the Hsp90-NTD and in red lines for the Cdc37-MD. The consecutive residue numbering is adopted and is consistent with the annotation in (A). The position of the lid motif and the peaks corresponding to functionally important residues are indicated by arrows and annotated. The position of the lid motif (residues 108–138) is highlighted and pointed to by oval circles surrounding the lid. (C) Structural mapping of the conformational mobility in the essential conformational space of the three lowest frequency modes. A surface-based protein representation is employed. The color gradient from blue to red indicates the decreasing structural rigidity of protein residues. The interfacial residues are shown colored spheres according to their mobility. The important functional residues are annotated according to their original crystallographic numbering. (D) The Hsp90-Cdc37 structure is annotated according to the adopted domain coloring. The lid motif in both structures is highlighted and colored in gold; the anchoring residues with the high force constants are indicated.

We also computed the NMSF and force constant profiles for the structurally different Hsp90-Cdc37 client recruiter complex. The mechanism of Cdc37-mediated inhibition of the ATPase activity is based on the hydrogen bonding between Cdc37-R167 and catalytic residue Hsp90-E47 that can prevent hydrolysis of ATP, although it could still allow for ATP binding [23]. According to the structural studies [23]–[25], the direct Cdc37 binding with the lid motif inhibits the formation of the closed lid conformation and triggers arrest of the Hsp90-ATPase cycle in the open Hsp90 conformation (Figures S1,S2). For the Hsp90-Cdc37 complex, the employed coarse-grained modeling approaches also converged to a consistent dynamics profile of the lid motif (residues 108–138 in the NMR structure [25]) demonstrating that structural immobilization of the lid is the fundamental dynamic feature of the Hsp90-Cdc37 binding (Figures 5A,C). In agreement with the experimental data [23]–[25], functional dynamics maps captured a more subtle effect by observing that the boundaries of the structurally stable core could be extended towards L29, A55, and L103 residues from the first, second, and fifth α-helices of the Hsp90-NTD (Figure 5A,C). The Cdc37 interfacial residues M164, L165, R166, R167, and L205 that displayed a strong decrease in signal intensity in the NMR experiments [29] were also structurally stable in the dynamics analysis. The force constant profile of the Hsp90-Cdc37 complex is marked by a steep hike for the lid residues reflecting a significant increase in structural rigidity (Figure 5B, D). Interestingly, the top 10% of high force constant residues in the Hsp90-Cdc37 complex include G132, Q133, V136, G137, and F138 residues from the lid motif (Figure 5B). Structural rigidity of the lid in Hsp90-Cdc37 complex determines the position of I110 and F138 hinge sites, clearly demarcating the borders separating structurally rigid core within the Hsp90-Cdc37 complex (Figure 5B, D). Hence, from a dynamic perspective, the primary inhibitory role of Cdc37 in arresting ATPase cycle may be fulfilled by switching conformationally mobile lid into “rigid” open position via local interactions and without invoking substantial allosteric changes. This mechanism is another manifestation of cochaperone-based manipulation of the lid dynamics. It is radically different from the Rar1-mediated mechanism that promotes the enhanced conformational mobility of the lid and effectively destabilizes both the fully open and fully closed lid forms.

Hence, coarse-grained dynamics analysis has identified common and distinctive dynamic signatures of the Hsp90-Sgt1 and Hsp90-Sgt1-Rar1 complexes as compared to the Hsp90-Cdc37 binding. Consistent with the experimental evidence, our results suggested that targeted modulation of the lid dynamics as a common characteristic of the client recruiter cochaperones. In summary, we provided a quantitative characterization of the functional dynamics in the Hsp90-cochaperone complexes that suggested a linkage between cochaperone-induced modifications of the lid dynamics and global structural changes that could enhance the ATPase activity. In the next section, we analyze networking characteristics of the Hsp90-cochaperone interactions to understand how targeted modulation of the chaperone dynamics is allosterically coupled with specific interaction networks that can inhibit or promote progression of the ATPase cycle and thus control the recruitment of diverse client proteins.

### The Rar1-CHORD2 Binding Stabilizes Network Communities in the Hsp90-Sgt1-Rar1 Complex

**Figure 6:**
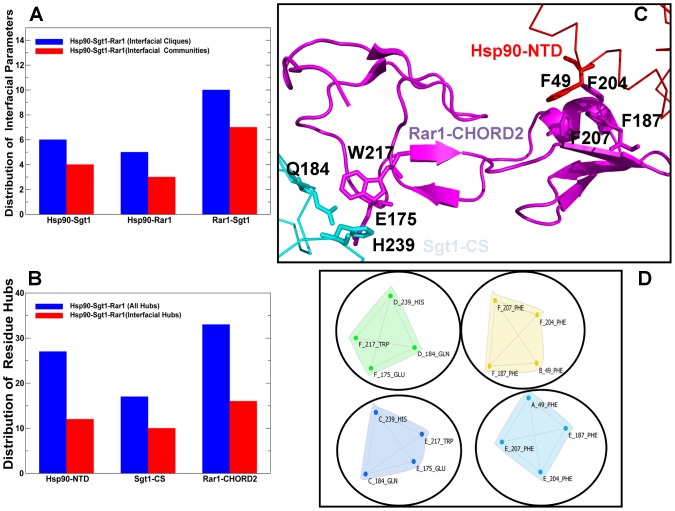
Network Analysis of the Hsp90-Sgt1-Rar1 Complex. (A) The distributions of the interfacial cliques (in blue filled bars) and the interfacial communities (in red filled bars) in ternary complex are shown respectively for the Hsp90-Sgt1, Hsp90-Rar1 and Rar1-Sgt1 binding interfaces. (B) The distribution of all residue hubs (in blue filled bars) and the interfacial hubs (in red filled bars) in the Hsp90-NTD, Sgt1-CS and Rar1-CHORD2 domains. (C) A close-up of structurally stable communities formed by the Rar1-CHORD2 residues with the Hsp90-NTD (Rar1-F187, Rar1-F204, Rar1-F207, Hsp90-F49) and the Sgt1-CS domain (Rar1-E175, Rar1-W217, Sgt1-Q184, Sgt1-H239). The interacting domains are annotated and shown in ribbons, Rar1-CHORD2 is colored in magenta, Hsp90-NTD is in red, and the Sgt1-CS is in cyan. The residues contributing to the interfacial communities are indicated. (D) The protein structure graphs of the major interfacial communities formed by the Rar1-CHORD2 domain in the heterohexamer Hsp90-Sgt1-Rar1 complex. The annotation of the interacting domains is consistent with Figure 3. The Hsp90-NTD domain (molecules A and B) interacts respectively with the Rar1-CHORD2 domain (molecules E and F). The Rar1-CHORD2 molecules E and F interact with the Sgt1-CS molecules C and D respectively. The protein structure graphs were obtained using the CFinder program [104].

We conducted a protein structure network analysis of the Hsp90-cochaperone complexes and analyzed principal differences in the interaction networks by evaluating the distribution of cliques, communities and hubs. These network parameters can characterize densely packed and structurally stable regions, thus providing a simple yet robust metric for evaluation of structural stability in the protein structures [80]–[87]. In the network analysis, communities were identified using both the interaction residue connectivity and cross-correlation contact maps [88] obtained from the ENM-based normal mode analysis. We focused on the network analysis of the regulatory Hsp90-Sgt1-Rar1 complex by placing a specific emphasis on the distribution of the interfacial cliques, communities and hubs (Figure 6A). It is evident that the Rar1-CHORD2 interactions in the ternary complex are central to the formation of the interaction network, producing a considerable number of interfacial communities. This analysis indicated that the Rar1-Sgt1 interactions resulted in the largest number of the interfacial communities, whereas the Rar1-Hsp90 and Sgt1-Hsp90 interactions could generate a similar and smaller number of such assemblies (Figure 6A). We also analyzed the distribution of hub residues in the functional complex, particularly the interfacial hubs that are connected with the residues at the intermolecular interface (Figure 6B). Interestingly, the Rar1-CHORD2 interactions could give rise to a significant number of the interfacial hubs that exceeded the contribution of both the Hsp90-NTD and Sgt1-CS domains. This distribution mirrors a similar trend in the organization of the interfacial cliques and communities. Consistent with structural and functional experiments [38]–[40], these results point to a critical role of the Rar1-CHORD2 interactions in the formation of the interaction network and stabilization of the Hsp90-Sgt1-Rar1 complex.

Structurally stable communities at the Rar1-CHORD2 interface are formed via cooperative interactions with the Hsp90-NTD and Sgt1-CS residues (Figures 6 C,D). In one of these communities Rar1-F187, Rar1-F204, and Rar1-F207 are interconnected with Hsp90-F49. Another prominent community is formed through the interactions of Rar1-E175 and Rar1-W217 with the Sgt1-CS residues Q184 and H239 (Figures 6 C,D). The interactions of Rar1-F187 support the proper positioning of the imidazole ring of Rar1-H188 interacting directly in the crystal structure with the β-phosphate of ADP in the Hsp90-NTD [40]. The experimental studies confer a broad functional role of the Rar1-CHORD2 residues F204, F207, and W217 involved in the formation of interaction communities. In particular, yeast two-hybrid assays have demonstrated that mutation of these Rar1 residues in the Rar1-CHORD2 domain only, but not in full length Rar1, substantially reduced the interaction with Hsp90 and destabilized the ternary complex [40]. According to these experimental studies, mutations of W217 could disrupt the interaction with Sgt1 in yeast two hybrid assay and *in vivo* co-immunoprecipitation assay. Moreover, these mutations are detrimental to the activity of the Hsp90-Sgt1-Rar1 complex by causing resistance to tobacco mosaic virus conferred by the NLR client protein [40].

**Figure 7:**
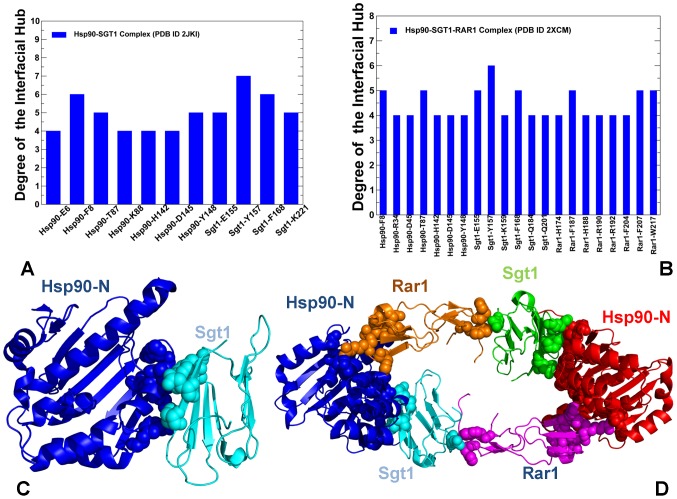
The Distribution of the Interfacial Hubs in the Hsp90-Sgt1 and Hsp90-Sgt1-Rar1 Complexes. The degree of the interfacial hubs in the Hsp90-Sgt1 complex (A) and Hsp90-Sgt1-Rar1 complex (B). The degree of a hub (or degree centrality) is the simplest measure of centrality and is defined as the number of links incident upon a node. The highly connected interfacial hubs with the number of connected residues exceeding the default threshold of four are shown for both complexes in filled blue bars. (C) Structural mapping of the interfacial hubs shown in (A) on the crystal structure of the Hsp90-Sgt1 complex (PDB ID 2JKI). The Hsp90-NTD is in blue ribbons, the Sgt1-CS domain is cyan ribbons. (D) Structural mapping of the interfacial hubs shown in (B) on the crystal structure of the Hsp90-Sgt1-Rar1 complex (PDB ID 2XCM). In the heterohexamer Hsp90-Sgt1-Rar1 complex, the two Hsp90-NTD molecules are shown in blue and red ribbons; the two Sgt1-CS domains are presented in cyan and green ribbons; and the two Rar1-CHORD2 domains are depicted in orange and magenta ribbons respectively. The interfacial residue hubs in (C) and (D) are mapped as spheres and annotated according to their domain color.

The protein structure network parameters could also provide a very approximate but simple measure for estimation of the binding energy changes in the Hsp90-cochaperone complexes. According to the relative number of structurally stable interfacial cliques and communities, the Rar1-Sgt1 binding should be considerably stronger that the Hsp90-Rar1 and Hsp90-Sgt1 interactions, suggesting that the RAR1–SGT1 interactions should play a key role in the stabilization and binding affinity of the ternary complex (Figures 6A,B). The community analysis of the Hsp90-cochaperone interactions is indeed consistent with the isothermal titration calorimetry (ITC) experiments that showed that the Kd value of the Rar1-CHORD binding with Sgt1-CS is 3.09 µM, which is appreciably lower than the Kd of 22.3 µM of the Hsp90-Rar1 binding and the Kd of 43 µM for the Hsp90-Sgt1 binding [40]. Overall, the community analysis is consistent with the structural and functional experiments [38]–[40], indicating the Rar1–Sgt1 interactions critically contribute to the stability of the regulatory ternary complex and, as such, may be of primary importance in the recruitment and activation of NLR client proteins. The interfacial hubs contribute to the stabilization of secondary structure elements within their own domains and integrate the cooperative interactions at the intermolecular interface. We then proceeded with a detailed characterization and comparison of the interfacial hub residues in the Hsp90-Sgt1 and Hsp90-Sgt1-Rar complexes (Figure 7). The residues in the same community are interconnected and can transfer the information through multiple routes, whereas there are typically fewer edges involved in the interaction between communities, and the nodes involved in this communication could be critical for allosteric interactions and for long-range signal transmission in the interaction network. We specifically focused on the distribution of highly connected interfacial hubs (with the number of connected residues exceeding the default threshold of four) in the binary and ternary complexes, since these hubs may reveal functional sites responsible for cochaperone-mediated regulation. In the binary Hsp90-Sgt1 complex the highly connected interfacial hubs in the Hsp90-NTD include critical residues F8, T87, K88, H142, D145, and Y148 (Figure 7A, C). Interestingly, we observed that the Sgt1-CS hubs have often a higher node degree that corresponds to a greater number of the interfacial neighbors. The most “influential” Sgt1-CS hubs include Y157 and F168 residues that are connected structurally (via interaction connectivity) and dynamically (by virtue of cross-correlated motions) with the significant number of residues (Figure 7A, C). These residues are involved in the core interactions that are primarily provided by the hydroxyl group of Sgt1-Y157 hydrogen bonding to the side-chains of Hsp90-K88. Additionally, the aromatic ring of Y157 forms the hydrophobic interactions with Hsp90-F8 and Hsp90-K88 [40]. In agreement with the structural analysis, Hsp90-F8 and Sgt1-Y157 residues also correspond to the peaks in the distribution of the interfacial hubs.

Not only our analysis correctly pinpointed to these residues as important interfacial hubs, but it also indicated that the key interactions formed by these residues may be supported via a dense network of additional contacts with the neighboring residues. These results pointed to the propensity of highly connected interfacial hubs to serve as functional hot spots of the Hsp90 activity. Indeed, targeted mutagenesis of the Hsp90-Sgt1 interface demonstrated that modifications of the Sgt1-CS residues Y157, F168, K221, and E223 would abrogate functional interactions and reporter activation [38]. All these residues, with the single exception of E223, emerged among highly connected network hubs (Figure 7). In this context, it is important to mention that though alanine mutations of Sgt1-E223 affected the Hsp90-Sgt1 interactions, they had a negligible functional effect on NLR client-mediated resistance to tobacco virus [38]. In contrast, mutations of the Sgt1-Y157 and charge reversals on the Sgt1-K221 sites resulted in a considerable functional effect. Hence, structure-based network analysis of the Hsp90-Sgt1 interactions revealed a small number of highly connected hubs which emerged as functionally important sites in a broad range of experimental investigations [36]–[40].

### Mapping Functional Dynamics Profiles of the Hsp90-Sgt1 and Hsp90-Sgt1-Rar1 Complexes with Network Parameters

By mapping network parameters onto the ENM-derived dynamics profiles in the essential conformational space, we could also characterize the role of specific residues in modulating structural stability of the regulatory complexes. The distribution of structurally stable communities is in good agreement with the population distribution profiles (Figures 2, 4). Strikingly, the noticeable peaks in the force constant profiles of the binary and ternary complexes (Figure 4) correspond to the Sgt1 residues Y157, F168, K221 that are among the highly connected interfacial hubs in the Hsp90-Sgt1-Rar1 complex (Figure 7 B,D). The Rar1-CHORD2 residues A185-H188 that displayed the higher force constants in the dynamic profiling of the Hsp90-Sgt1-Rar1 complex (Figure 4B) were also among notable hubs at the intermolecular interface (Figure 7 B, D). Moreover, these residues also contribute to the interfacial communities and are central to the stabilization of the interaction network in the functional complex. According to our model, the highly connected interfacial hubs are located within dense protein regions having more interacting neighboring nodes than a typical residue in the Hsp90-cochaperone complex. Not only the Rar1 interfacial hubs at the Hsp90-Rar1 binding site have a large number of neighbors but they are also supported by other well-connected residues, thus leading to a dense structural core of stable and interconnected residues (Figure 7B,D). These findings are consistent with the notion that different networks (including protein structure networks and protein-protein interaction networks) are often formed via overlapping modules and could exhibit a hierarchical organization where small, highly connected modules (communities) could associate into larger units [101]–[104].

We observed that the protein structure networks of the Hsp90-cochaperone complexes may have some elements of a hierarchical structure, in which central highly connected hubs are locally surrounded and interact with less important hubs that have fewer interacting neighbors. As a result, targeted perturbations of highly connected hubs could simultaneously disrupt many interactions leading a significant loss in chaperone activity. The dual role of these residues in anchoring functional motions and promoting stabilization of the ternary complex may explain some of the experimental observations which have emphasized the importance of these hot spots to the activity of the Hsp90-cochaperone complex [38]–[40]. It is worth noting that flexible lid residues are conspicuously absent in the structurally stable communities and are not capable of serving as the network hubs. Hence, the exceedingly mobile lid motif could be decoupled from the stable interfacial communities, thus permitting its free excursions between the open and closed lid forms without perturbing the interaction network.

In summary, we found that (a) the largest number of the interfacial hubs is in the Rar1-CHORD2 domain, and (b) the incorporation of the Rar1-CHORD2 domain led to the increased number of hubs in the Sgt1-CS domain (Figure 7). At the same time, the interfacial hubs from the Hsp90-NTD are confined to the Hsp90-Sgt1 interface. These results suggested that the Rar1-Sgt1 interactions form the central core of the interaction network that could stabilize the ternary complex. The most connected interfacial hubs in the Hsp90-Sgt1-Rar1 complex include Sgt1-Y157, Sgt1-F168, Rar1-F187, Rar1-F207, and Rar1-W217 (Figure 7B, D). It is evident from this analysis that a limited set of key residues may critically contribute to the thermodynamic stability of the functional complex. We also observed that the hub residues contribute to the formation of cliques and communities which assures the cooperativity of the intermolecular interactions. In particular, the Rar1-CHORD2 residue hubs F187, F204, and F207 are interconnected with each other in the interfacial communities (Figure 6). Hence, the organization of the interaction network in the functional complex appeared to be strongly influenced by the presence of the Rar1-CHORD2 domain and largely determined a selected group of highly connected Rar1 residues. This may transpire in the denser network of interconnected nodes in the Hsp90-Sgt1-Rar1 complex as compared to the Hsp90-Sgt1 complex, and thus facilitate cooperative interactions in the functional assembly.

### Protein Network Analysis of the Hsp90-Cdc37 Interactions

**Figure 8:**
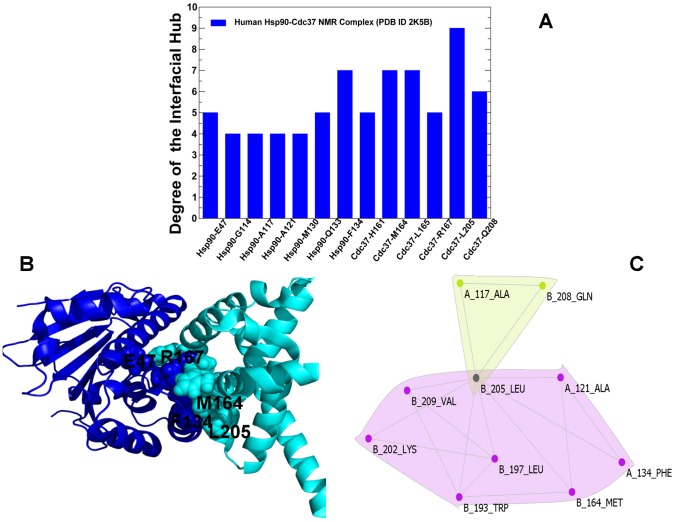
The Distribution of the Interfacial Hubs in the Hsp90-Cdc37 Complex. (A) The degree of the interfacial hubs in the Hsp90-Cdc37 complex. The highly connected interfacial hubs with the number of connected residues exceeding the default threshold of four are shown for both complexes in filled blue bars. (B) Structural mapping of the interfacial hubs shown in (A) on the NMR structure of the Hsp90-Cdc37 complex (PDB ID 2K5B). The Hsp90-NTD is in blue ribbons, the Cdc37-MD is cyan ribbons. (C) The protein structure graphs of the major interfacial communities formed by in the Hsp90-Cdc37 complex. The graph illustrates the key role of the Cdc37-L205 residue hub in integrating a number of stable communities in the Hsp90-Cdc37 complex. The protein structure graphs were obtained using the CFinder program.

We also compared the organization of the interaction networks between structurally different Hsp90-Sgt1-Rar1 and Hsp90-Cdc37 complexes to probe general principles of the Hsp90 interactions with client recruiter cochaperones. This analysis highlighted a common role of the interfacial hubs as functional regulators of the Hsp90 activity. The highly connected hubs in the Hsp90-NTD corresponded to the M130, Q133, and F134 residues. The Cdc37-MD hubs that contribute to the structural stability of the Hsp90-Cdc37 complex included M164, L165, R167, L205 and Q208 residues (Figure 8A). We also noticed a good correspondence between the dynamic force constant profiling and distribution of the hub residues in the interaction network. Of special interest the appearance of key functional Cdc37 residues R167 and L205 as prominent peaks in the force constant profile (Figure 5B). Concurrently, these residues emerged as highly connected hubs in the structural network, where L205 residue is locally connected to the total of nine neighboring residues from both interacting proteins (Figures 8A, B). Interestingly, L205 contributes to the formation of several stable communities within the Cdc37-MD as well as the interfacial communities formed with the partnering Hsp90-NTD residues A117, A121, F134 (Figure 8C). The prominent communities in the Hsp90-Cdc37 complex are all anchored by L205 as a central residue, including the stabilizing intradomain community of Cdc37-MD residues W193-L197-K202-L205-V209 as well as the key interfacial community composed of the Hsp90-NTD residues A121 and F134 that are interconnected with the Cdc37-MD residues M164 and L205 (Figure 8C). The graph-based representation could illustrate the hierarchical nature of the interaction connectivity in the Hsp90-Cdc37 complex that is marked by the emergence of the central integrating residue hub L205 surrounded by multiple layers of supporting residues and smaller communities that create the complex interconnected web. Despite the simplicity of the network-based analysis, the results are in an excellent agreement with the NMR experiments [25] that implicated L205 as a single key residue enabling the complex formation. We also observed that a small number of Cdc37-MD residue hubs may provide a decisive contribution to the Hsp90-Cdc37 binding and stabilization of the regulatory complex.

These results are also consistent with the detailed biochemical analysis of the full-length human Hsp90-Cdc37 complex [105] that identified critical residues and their contributions to the Hsp90-Cdc37 interactions in living cells. According to these experiments, mutations in the Hsp90-NTD (Q133A, F134A, and A121N) and mutations in the Cdc37-MD (M164A, R167A, L205A, and Q208A) could dramatically reduce the Hsp90-Cdc37 interactions by as much as 70–95%. Although the extensive hydrophobic interface with the large buried surface area (∼1600 Å2) is formed in the NMR structure of the Hsp90-Cdc37 complex [25], single mutations in selected number of residues could be sufficient to disrupt the Hsp90-Cdc37 complex and displace Cdc37 from the chaperone system [105]. The network analysis of the Hsp90-Cdc37 complex recapitulated these central experimental findings. Indeed, Q133, F134, M164, L205, Q208 were found among the most connected interfacial hubs, where F134, M164, L205 are directly connected to 7–9 residue nodes in the interaction network (Figure 8). As a result, the alanine mutations of these residues could simultaneously abolish many favorable interactions and disrupt the organization of the interaction network, leading a significant loss in binding and cochaperone activity [105]. Moreover, the degree of the hub connectivity correlates with the contributions of critical residues in the Hsp90-Cdc37 binding that were experimentally ranked in the following order Q133> F134> E47 [105]. Indeed, we found that these residues correspond to the major interfacial hubs, where Q133 and F134 have the largest number of local interacting neighbors (Figure 8). Hence, Cdc37-mediated inhibition of the ATPase activity is determined by the interaction network of a small number of specific hydrophobic and polar residues that can stabilize the open lid conformation and arrest the progression of the ATPase cycle.

### The Centrality Analysis of the Interaction Networks: Global Mediators of Long-Range Communications Are Functional Hotspots of Hsp90 Regulation

**Figure 9:**
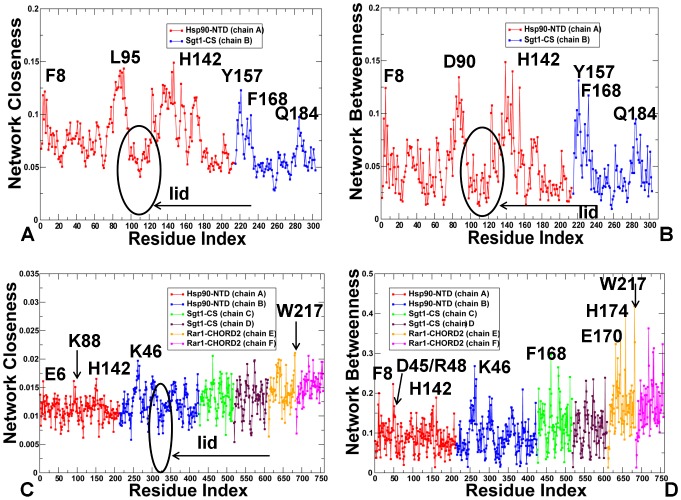
The Centrality Analysis of the Interaction Networks in the Hsp90-Sgt1 and Hsp90-Sgt1-Rar1 Complexes. The residue-based centrality parameters: closeness (A, C) and betweenness (B, D) of the Hsp90-Sgt1 and Hsp90-Sgt1-Rar1 complexes. In (A) and (B) the centrality profile for the Hsp90-NTD residues is shown in red lines, and for the Sgt1-CS residues in blue lines. In (C) and (D) the centrality profile for the Hsp90-Sgt1-Rar1 residues is also colored according to their domain annotation. The Hsp90-NTD molecules are in red/blue; the Sgt1-CS molecules in green/brown; and the Rar1-CHORD2 molecules in orange/magenta. The peaks in the centrality profiles corresponding to the functional hot spot residues of the Hsp90 activity are annotated and pointed by arrows. The lid residues in the Hsp90-Sgt1 and Hsp90-Sgt1-Rar1 complexes are highlighted and pointed to by oval circles surrounding the lid. To streamline the comparison with structural and functional experiments, we annotated functional residues according to their original numbering in the crystal structures.

We have thus far utilized the simplest measure of centrality, which is the degree centrality defined as the number of interacting residues that a particular node is connected to. However, the degree of a node could identify only locally connected hubs that are determined mainly by the local structural environment. This measure does not consider the global structure of the interaction network, i.e. although a node might be connected to many local neighbors, it might not be strategically positioned to quickly reach other residues in the network. Here, we explore weighted graph parameters such as node closeness and betweenness to determine globally connected nodes that could mediate efficient long-range communication between residues in the protein network. We evaluated the propensity of protein residues to serve as mediator nodes by computing the closeness and betweenness indices and focusing on the peaks in the respective centrality profiles as indicators of key mediating residues in the network (Figure 9). The closeness is a natural distance metric between all pairs of nodes and measures the inverse of the average of the shortest path between a residue and all other residues. Betweenness centrality quantifies the number of times a node could act as a bridge along the shortest path between any other two nodes. Due to their structural position, residues corresponding to the peaks in the centrality profiles are coupled to the other residues in the network over long-range distances and could mediate long-range allosteric interactions.

The centrality analysis of the Hsp90-Sgt1 (Figure 9A, B) and Hsp90-Sgt1-Rar1 complexes (Figure 9 C, D) revealed important differences in the distribution of mediator residues. We observed that the closeness of the Hsp90-NTD residues in the binary complex (Figure 9A) was somewhat higher than for the Sgt1-CS residues. On average, both closeness and betweenness indices were roughly similar for both domains (Figure 9A, B). The high peaks in the centrality profiles were consistent using both metrics and reflected the mediating role of the Hsp90-NTD residues F8, L95 and H142 as well as Sgt1-CS residues Y157, F168, and Q184. Importantly, these interfacial residues also emerged as prominent peaks in the force constant profiling (Figure 4) and were detected as highly connected local interaction hubs (Figure 7). Hence, a selected group of functional residues may serve as both local and global mediators in the interaction network of the Hsp90-Sgt1 complex (Figures 9A, B). The distinctive feature of the Hsp90-Sgt1-Rar1 complex is the characteristically higher value of the closeness and betweenness for the Rar1-CHORD2 residues in the ternary complex as compared to the Hsp90-NTD and Sgt1-CS domains (Figures 9C,D). According to our findings, a panel of selected Rar1-CHORD2 residues displayed the highest network centrality in the ternary complex. The Rar1-CHORD2 residues corresponding to the notable peaks in the closeness (and betweenness) profiles are E170, H181, K195 and W217, with the highest peak in both Rar1 molecules mapped precisely onto the position of the critical mediating residue W217 (Figures 9C,D). These residues serve as central mediators of the global interaction connectivity and thus are vital to the regulatory function of the ternary complex. Not only a selected group of the Rar1-CHORD2 residues displayed the highest network centrality in the ternary complex, but also the entire Rar1-CHORD2 domain seems to be involved in supporting these key nodes by exhibiting above average values of closeness and betweenness in the complex (Figures 9C, D). Furthermore, the Rar1-CHORD2 binding may lead to a partial rewiring of the functional residues in the ternary complex. Indeed, the peaks in the centrality profiles for the Hsp90-NTD residues shifted in the ternary complex towards D45, K46, and R48 that are involved in the Hsp90-Rar1 interactions (Figures 9C, D). In other words, the Rar1-CHORD2 binding may induce the reorganization of the Hsp90 mediating residues that would be primarily assembled at the Hsp90-Rar1 interface rather than in the Sgt1 interacting site. Since the structural mode of Hsp90-Sgt1 interaction is unaffected by the Rar1-CHORD2 binding, the Sgt1 mediating residues from the Hsp90-Sgt1 interface are preserved in the ternary complex. More importantly, the average closeness and betweenness of the Sgt1-CS residues noticeably increased in the ternary complex (Figures 9C, D), suggesting that Rar1 binding may enhance the contribution of the Sgt1-CS domain to the allosteric interactions. In general, because of the increased number of Rar1-CHORD2 nodes with higher closeness/betweenness, the average minimal distance between any pair of nodes in the Rar1-CHORD2 domain would be distinctly smaller than in other domains, suggesting a central role of Rar1-CHORD2 in mediating allosteric communications in the ternary complex. Consistent with functional studies [38]–[40], this network-based assessment of mediating capabilities in the Sgt1-CS suggests that Rar1-CHORD2 would not interfere but rather stabilize the Hsp90-Sgt1 binding interactions, i.e. Rar1 would act as an enhancer of the Hsp90-Sgt1 chaperone system.

Mapping of the conformational mobility profiles with centrality parameters could also help to explain an apparent dilemma in which Rar1 is capable of enhancing the Hsp90-ATPase activity while destabilizing the active closed lid conformation [32], [40]. The globally connected mediating sites corresponding to the peaks in centrality profiling correspond to structurally stable regions in the essential conformational space of principal motions. This reflects the fact that more central amino acids (having a shorter average of their shortest path lengths) have a more restricted motion.

The centrality profiles revealed that, unlike highly connected mediating sites, the lid residues exhibit low closeness and betweenness values. The low mediating capabilities of most lid residues could arise from their high mobility and weak coupling with the structural core of the functional complex. Hence, the Rar1-CHORD2 binding may accelerate free conformational movements of the lid, thus effectively destabilizing both the lid-open ADP-bound and lid-closed ATP-bound conformations. Based on this model, it could be dynamically feasible for RAR1 to divert the Hsp90-ATPase conformational cycle from reaching the closed dimerized state, while allowing the catalytic residue to transiently access the nucleotide site and promote ATP hydrolysis. This model is consistent with the proposed mechanism of Rar1-mediated regulation of the ATPase activity [38]–[40].

The network-based centrality analysis may help to rationalize several subtle functional experiments, particularly the fact that some mutants of Rar1-W217 that abrogate the interactions with Sgt1-CS could still bind Hsp90-NTD in the absence of Sgt1-CS, but the interaction with Hsp90 would be greatly decreased in the presence of Sgt1-CS [40]. Indeed, according to our results, the community formed by W217 at the Rar1-Sgt1 interface may be allosterically coupled through abundance of mediating routes with another community of the Rar1 interfacial hubs (F187, F204, and F207) at the Rar1-Hsp90 interface. Although the residues within each of these communities are strongly interconnected, only a few highly globally mediating nodes are primarily responsible for long-range signal transmission between remote communities. According to our findings, the Rar1-CHORD2 residues E170, F187, F204, F207, and W217 could form a major bottleneck for the information transfer in the network. The Rar1-CHORD2 domain harbors the critical functional sites, including E170 and W217 at the Rar1-Sgt1 interface, that serve not only as highly connected local interaction hubs but also as global mediators of allosteric communication in the ternary complex (Figures 9C,D). In addition, the average centrality of the Rar1-CHORD2 residues is higher than in the other domains, i.e. the key mediator residues are surrounded by “supporting cast” residues that have sufficient communication capacity to rapidly disseminate the information signal from central mediators across the network. Hence, the Rar1-CHORD2 binding could regulate the long-range interactions and promote stabilization of the ternary complex. The network analysis could thus clarify the elusive role of Rar1 as a key component of the ternary assembly and stability enhancer of the Hsp90-cochaperone interactions [38]–[40]. Overall, our results support the mechanism in which a selected group of critical Rar1-CHORD2 residues, particularly Rar1-W217 as a potential principal contributor, may be critical for mediating long-range interactions and modulation of the Hsp90-ATPase activity. Our results indicated that the Rar1-CHORD2 binding may result in a more assortative interaction network [106], [107] that is better integrated through preferential association of global mediating residues with many locally connected hubs.

**Figure 10:**
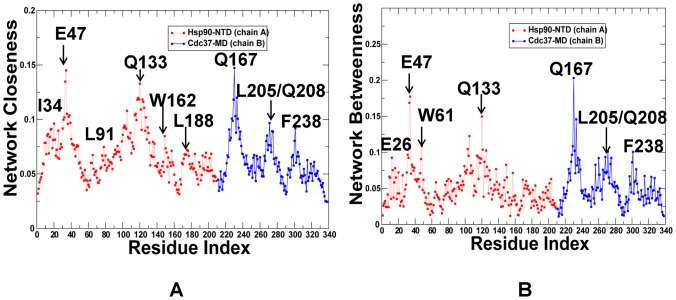
The Centrality Analysis of the Interaction Networks in the Hsp90-Cdc37 Complex. The residue-based centrality parameters: closeness (A) and betweenness (B) of the Hsp90-Cd37 complex. The centrality profile for the Hsp90-NTD residues is shown in red lines, and for the Cdc37-MD residues in blue lines. The consecutive residue numbering of the Hsp90-NTD and Cdc37-MD residues is adopted as in Figure 5. The original numbering of the Hsp90-NTD (residues 14–223) in the NMR structure corresponds to residues 1–210, and the original numbering of the Cdc37-MD (residues 148–276) corresponds respectively to residues 211–339. The peaks in the centrality profiles corresponding to the functional hot spot residues of the Hsp90 activity are annotated and pointed by arrows. The lid residues in the Hsp90-Sgt1 and Hsp90-Sgt1-Rar1 complexes are highlighted and pointed to by oval circles surrounding the lid. To facilitate the comparison with structural and functional experiments, we annotated functional residues according to their original numbering in the crystal structures.

The centrality analysis of the Hsp90-Cdc37 complex similarly revealed that key mediating residues and functional hot spots are aligned with the peaks in the closeness (Figure 10A) and betweenness profiles (Figure 10B). The characteristic peaks were conserved in both distributions and corresponded in the Hsp90-NTD to the residues E47 and Q133. Both residues form stabilizing polar interactions with R167 in Cdc37 and these interactions are implicated as a major contributing factor in the mechanism of Cdc37-mediated inhibition of the ATPase activity [23]–[25], [105]. The centrality analysis recovered major functional sites E47, Q133, and F134 as key mediating residues of allosteric communications in the Hsp90-Cdc37 complex. Interestingly, E47A mutation reduced Hsp90-Cdc37 binding by 50%, while Q133A mutation could decrease the Hsp90-Cdc37 interactions by 85% [105]. While our analysis robustly selected the Hsp90 binding hot spots, the simplicity of the centrality metric somewhat underestimated the relative contribution of Q133 to the Hsp90-Cdc37 binding that is more significant that was predicted. In the Cdc37-MD, the centrality peaks corresponded to R167 and Q208 residues (Figure 10) that were also among highly connected interfacial hubs. Overall, the centrality analysis suggested that the group of strongly interacting residues Hsp90-E47, Hsp90-Q133, Cdc37-R167, and Cdc37-Q208 could collectively form a major gateway for allosteric communications in the Hsp90-Cdc37 complex (Figure 10). These global mediating residues are also highly connected local hubs and major contributors of the structurally stable communities. The prominent role of these residues in the Hsp90-Cdc37 binding was demonstrated via a comprehensive biochemical analysis [105] in which mutations of Hsp90-Q133, Cdc37-R167 and Cdc37-Q208 essentially abolished the complex formation, causing 85%–90% reduction in the Hsp90-Cdc37 interactions as measured in cell-based assays. These key functional sites were consistently recovered as largest peaks in both closeness and betweenness metrics. Mutations of key mediating nodes in the Hsp90-Cdc37 complex would cause a simultaneous disruption of multiple interactions and disrupt the integrity of the allosteric network, thus leading to a dramatic loss of the chaperone activity. At the same time, the centrality analysis unveiled a few smaller noticeable peaks corresponding to the Hsp90-NTD residues Y61, L91 W162 and Cdc37-F238 (Figure 10). These residues are not located at the interdomain interface and reside within their respective domains, contributing to the structural integrity of the interacting modules. Although these residues may contribute to the intradomain communications, their role as global mediating nodes of long-range communications is less prominent and respectively their mutations have a smaller effect on the ATPase activity [105].

### Small-world Organization of the Hsp90 Interaction Networks

We argue for the functional relevance of the centrality parameters as robust indicators of functionally important sites as this network analysis captured most of the known loss-of-function mutations [105]. The organization of the interaction networks in the studied Hsp90-cochaperone complexes gives rise to small-world networks, marked by a relatively small number of highly connected mediators occurring mostly at the intermolecular interfaces and playing critical roles in the transmission of functional signals. Small-world networks are characterized by small separation of nodes from each other, which for proteins means a higher degree of interaction cooperativity. The vulnerability of the interaction networks to targeted perturbations of highly connected hubs may explain why mutations of these critical hot spot residues could lead to a significant loss in chaperone activity. Complex networks may be either disassortative (links between hubs are systematically suppressed) or assortative (links between hubs are enhanced) [106]–[110]. In the network analysis, disassortativity produces better connected but more vulnerable networks, whereas assortativity gives rise to more resilient networks. We found that the interaction networks of the Hsp90-cochaperone complexes may undergo a specific rewiring of key mediating residues and assortative growth as a result of conformational equilibrium changes during protein-protein binding. Our results also suggested that the interaction networks may evolve the network of “supporting” residues that acquire sufficient communication capacity to pass signal from various central mediators across the network.

The evolution of various networks has been extensively studied and a scale-free model of network organization has gained a considerable recognition [102], [103], [111], [112]. This model is based on the idea of “preferential attachment” where the most connected nodes are more likely to acquire new edges in the course of graph evolution. The scale-free topology of a network arises from network growth and preferential attachment endowing the network with high efficiency and robustness against random errors due to a small number of central nodes and exceedingly large number of peripheral nodes. However, due to the finite size of the protein structure graphs and topological constraints, the degree distribution of protein structure networks do not follow the preferential attachment scenario and is not scale-free, but is likely to be Poissonian [113]–[117]. The fundamental reason for deviation from the scale-free behavior is the inherently limited interacting capacity of a given residue within a structural fold due to the excluded volume effect. The scale-free networks are highly efficient in transmitting long-range signal due short paths between any pair of nodes, but could be extremely vulnerable to targeted attacks on a few key hubs that could result in splitting the global network into smaller pieces. We suggest that protein structure networks may be more tolerant to targeted attacks at the expense of some efficiency by virtue of creating broad-scale connectivity in which global hubs and central mediators are often protected by a dense network of “secondary” hubs that could mitigate the effect of targeted mutations in certain functional sites. A possible explanation for the functional lethality of some, but not all, mediating residues is that assortative hubs are likely to be responsible for long-range communications that may be compromised by deleterious mutations. Although mutations of these residues may often result in a dramatic loss of activity, some of these alterations could be rescued by the presence of well-connected supporting hubs that may assume “responsibility for global centrality” in the mutation-damaged interaction network.

Our results argue for relevance of the synergistic approach that combines functional dynamics and protein structure network analyses as a simple yet robust tool for probing subtle mechanisms of Hsp90 regulation by cochaperones and client proteins. However, computational modeling presented in this study critically depends on the availability of structural information about Hsp90-cochaperone complexes. A critical question remains is how the Hsp90-Sgt1-Rar1 system can ensure selective recruitment of NLR proteins and whether the chaperone machinery is designed to recognize a recognition-competent folded state or NLR proteins undergo folding upon binding scenario. Dissecting the intricate interaction network that connects Hsp90, Sgt1, Rar1 and NLR proteins is a prerequisite to further capture the specificities of this important client family. Structural and computational challenges of quantifying the Hsp90 interactions with the vast array of interacting proteins are especially relevant in light of recent proteomics studies that have suggested how structural diversity of the Hsp90 clientele may be handled by the chaperone machinery [118]–[121]. This analysis has clarified the mechanism of Hsp90 client recognition as a two-step process: its co-chaperones provide specificity at the protein-fold level, whereas thermodynamic parameters determine client binding within a protein family. According to these studies, the critical determinants that control recognition and recruitment of client proteins include preferential binding to the clients that are only marginally stable in their native folds. In particular, Hsp90-kinase binding is sensitive to the conformational status of the kinase as strong clients of Hsp90 are intrinsically unstable kinases. Consequently, allosteric stabilization of the kinase fold by small molecules strongly decreases chaperone interaction, suggesting that the Hsp90 chaperone could serve as a “thermodynamic sensor” of drug-protein interactions in living cells [119]. The integration of structural, functional and computational studies of the Hsp90 interactions would likely to be useful for probing mechanisms of molecular chaperones and guiding discovery of allosteric Hsp90 modulators.

## Conclusions

By combining functional dynamics and protein structure network analyses, the reported study integrated structural bioinformatics and molecular simulation approaches to dissect complex mechanisms of Hsp90 regulation. Distinctive dynamic and networking signatures of the Hsp90-Sgt1 and Hsp90-Sgt1-Rar1 complexes have demonstrated how targeted modulation of the lid dynamics is coupled with specific interactions that can inhibit or promote progression of the ATPase cycle. We proposed a model in which cochaperone-mediated regulation of the lid dynamics could be reminiscent of a rheostat mechanism that adjusts the lid mobility to induce the required changes in the ATPase activity. In this mechanism, the Hsp90-cochaperone system may successfully bypass stochastically-driven slow conformational changes of the Hsp90 dimer and facilitate ATP hydrolysis.

Protein network analysis of the Hsp90-cochaperone interactions has also identified structurally stable interaction communities, interfacial hubs and key mediating residues of allosteric communication pathways. The results have shown that client recruiter cochaperones can orchestrate global changes in the dynamics and stability of the interaction networks that could enhance the ATPase activity and assist in the client recruitment. The network analysis has reproduced a number of structural and mutagenesis experiments, suggesting that the network parameters and centrality analysis could present a robust and simple tool for predicting hot spots of the Hsp90 activity.

Small-world organization of the interaction networks in the Hsp90 regulatory complexes gives rise to a strong correspondence between highly connected local interfacial hubs, global mediator residues of allosteric interactions and key functional hot spots of the Hsp90 activity. The vulnerability of the interaction networks to targeted perturbations of highly connected hubs may explain why mutations of these critical hot spot residues could simultaneously disrupt many interactions leading a significant loss in chaperone activity. The results of this study suggest that the topology of the interaction networks may be determined by the structural architecture of the Hsp90 complexes and functionally important changes that direct the ATPase cycle are often coordinated by a small number of highly connected conserved hubs.

## Materials and Methods

### Coarse-Grained Modeling of Functional Dynamics

The functional dynamics analysis of the Hsp90-cochaperone complexes was conducted using the GNM approach [41]–[43] in which protein structure is reduced to a network of *N* residue nodes identified by 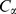 atoms and the fluctuations of each node are assumed to be isotropic and Gaussian. The topology of the protein structure is described by N × *N* Kirchhoff matrix of inter-residue contacts 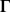, where the off-diagonal elements are −1, if the nodes are within a cutoff distance 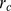, and zero otherwise. Bonded and nonbonded pairs of residues located within an interaction cutoff distance 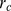  = 7.0 Å are assumed to be connected by springs with a uniform spring constant γ. The equilibrium dynamics of the structure results from the superposition of *N*−1 nonzero modes found by the eigenvalue decomposition of 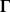. The GNM-derived cross-correlations between the fluctuations 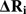 and 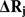 of residues (nodes) *i* and *j* were also computed. The normal modes are found by diagonalization of the Kirchhoff matrix 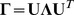.
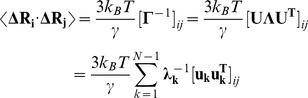
(1)Here, 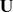 is a unitary matrix, 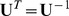 of the eigenvectors 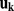 of 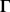 and 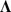 is the diagonal matrix of eigenvalues 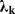.The elements of the *k*th eigenvector 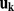 describe the displacements of the residues along the *k*th mode coordinate, and the *k*th eigenvalue, 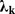, scales with the frequency of the *k*th mode, where 1≤ *k* ≤ *N* –1.

The normal mode analysis generated eigenvalues 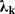 and eigenvectors 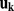 obtained using the WEBnm@ approach [122]. The normal mode analysis included computation of 20 low frequency modes using an approximation that was proven to be highly efficient and accurate in describing functional protein motions [123].

The root mean square fluctuations of a given residue can be then evaluated as a sum over the contributions of all modes. The fluctuation of the *i* ^th^ atomic degree of freedom along the eigenvector 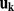 reflects the mobility of residue *i* in the *k*th mode.
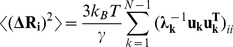
(2)

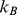
 is Boltzmann constant, the temperature T in the simulation is 300 K and N is the number of considered normal mode. The residue-based fluctuations and conformational mobility profiles were estimated using oGNM computation of structural dynamics based on the GNM approximation [43].

### Discrete Molecular Dynamics and Force Constant Analysis

We explored the force constant method [96] using the formalism of the discrete molecular dynamics (dMD) simulations [97]–[99] as implemented in [100]. According to the dMD approach, the protein structures were modeled as systems consisting of 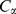 residue-based beads interacting through a discontinuous square well potential. In the basic dMD formalism [98], [99] particles move in the ballistic regime under constant velocity until a collision between a pair of particles occurs. In the absence of any collision, the particles move linearly with constant velocity.

The interaction potentials are defined as infinite square wells, such that the particle-particle distances vary between 
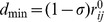
 and 
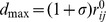
 where 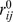 is the distance between particles (residues) 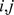 in the native conformation and 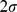 the width of the square well. The MD-averaged conformation was taken as the native conformation. Residue-residue interaction potentials are defined for the particles at a distance smaller than a cut-off radius 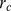 in the native conformation. A small well width 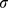 = 0.05 was used for neighboring particles to keep the 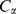
–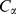 distances closer to the expected equilibrium value of 3.8 Å. For nonconsecutive pairs of 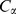 particles, 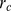 = 8 Å and 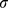 = 0.1 were used.

The dMD trajectories were used to compute the force constant values for each residue as was originally introduced in [96] and implemented in the FlexServ web-based tool [100]. A force constant for each residue is calculated by averaging the distances between the residues over the dMD trajectory using the following expression:
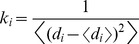
(3)

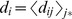
(4)


Here 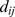 is the instantaneous distance between residues 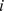 and 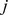. In accordance with the original definition [96], 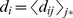 corresponds to the average distance between residues 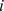 to all other residues in the protein. The interactions between the 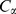 atom of residue 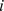 and the 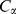 atom of the neighboring residues 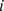
−1 and 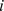
+1 are excluded in the calculation. The ignored interactions correspond to largely constant distances between consecutive particles.

### Protein Structure Network Analysis

The network analysis of the Hsp90-cochaperone complexes was conducted by generating graphs in which amino acid residues were considered as nodes connected by edges corresponding to the noncovalent interactions. The noncovalent interactions between side chain atoms are included and the interactions between sequence neighbors are ignored. The pair of residues with the interaction strength 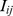 greater than a user-defined cut-off (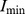) are connected by edges and produce a protein structure network graph for a given interaction strength 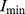. In accordance with the analyses of protein structure [80], [84], the optimal interaction strength 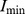 is typically in the range 2–4% for most of the protein structures. We considered any pair of residues to be connected if 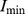 was greater than 3.0%.

The analysis of the interaction networks was done using network parameters such as hubs, cliques and communities. The hubs are highly connected nodes in the network. If the total number of edges incident on the node (called the degree of a node) is at least 4 the node is identified as a hub. The 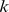-cliques are complete sub graphs of size k in which each node is connected to every other node. In our application, a 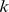-clique is defined as a set of 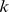 nodes that are represented by the protein residues in which each node is connected to all the other nodes. A 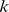-clique community is determined by the Clique Percolation Method [104] as a subgraph containing 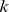-cliques that can be reached from each other through a series of adjacent *k*-cliques. We have used a community definition [80]–[87] according to which in a 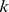-clique community two 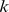-cliques share 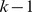 or 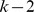 nodes. Community size is determined by the number of constituent cliques and is typically proportional to the compactness in the proteins. The construction of protein structure graphs was done with the web-based tool that converts protein structures into graphs (http://vishgraph.mbu.iisc.ernet.in/GraProStr/). Computation of the network parameters was performed using the Clique Percolation Method as implemented in the CFinder program [104]. Given the chosen interaction strength 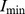 we typically obtain communities formed as a union of 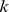 = 3 and 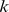 = 4 cliques.

Similarly to the original approach introduced by Luthey-Schulten and co-workers [88] we also employed the weighted network representation that combines the non-covalent connectivity of side chains and ENM-derived residue cross-correlation information in the construction of graphs. This protein network model is described by the weighted graphs where the weight 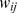 of an edge between nodes 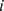 and 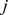 measures the cross-correlation dynamic connectivity of the nodes connected by that edge. The weight 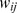 is defined as 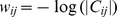 where 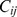 the cross-correlation value between the residues is 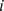 and 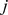 which is obtained from the ENM-derived normal mode analysis of the Hsp90-cochaperone complexes.

### Centrality Analysis: Closeness and Betweenness of the Interaction Networks

Using the constructed protein structure networks, we also computed the global centrality measures such as closeness and betweenness. Central to the computation of these parameters is the determination of the shortest paths between two given residues. Closeness centrality is defined as the inverse of the average of the shortest path between a residue 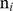 and all other residues:
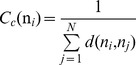
(5)


And the normalized closeness centrality is expressed as:
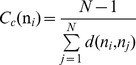
(6)


Betweenness centrality quantifies the number of times a node 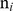 acts as a bridge along the shortest path between two other nodes. The betweenness measures the frequency of a given residue to belong to all shortest path pairs within the protein structure:
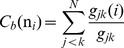
(7)where 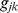 denotes the number of shortest geodesics paths connecting *j* and *k*, and 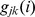 is the number of shortest paths between *j* and *k* passing through the node 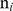. In the normalized representation, betweenness centrality expressed as
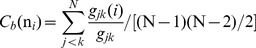
(8)where 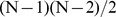 is the number of pairs of vertices (nodes) excluding the given node. The shortest paths between two residues are determined using the Floyd–Warshall algorithm that compares all possible paths through the graph between each pair of vertices. Network graph calculations were performed using the python module Network [124]. The characteristic path length (CPL), defined as an average of the shortest path length between all pairs of nodes in the network, provides an estimate of the effect of node connectivity on communication pathways in a protein.

## Supporting Information

Figure S1 **The Hsp90-Cdc37 Chaperone Cycle.** Clockwise from top left, ATP binding to the Hsp90-NTD of the apo Hsp90 in the open form induces a fast dynamic exchange between a nucleotide-free Hsp90 and an intermediate ATP-bound state in which the ATP lids and Hsp90-NTDs are still open. The kinase-specific cochaperone Cdc37 acts early in the chaperone cycle and binds to the Hsp90-NTDs inducing a partially contracted open form of Hsp90 and stabilizing the nucleotide-free chaperone conformation. Protein kinase clients are recruited to the Hsp90 system though the action of Cdc37. After ATP binding, Hsp90 reaches the next intermediate state in which the ATP lids are closed but the Hsp-90-NTDs are still open. Starting from Cdc37-Hsp90 complexes, ATP binding results in an open-closed equilibrium. The hydrolysis is inhibited by Cdc37. In an intermediate ATP-bound complex, with the ATP lids closed but the Hsp90-NTDs are still separated, the cochaperone Aha1 binds to the Hsp90-MD via its N-terminal domain and begins to compete with Cdc37 binding. ATP binding shifts the binding properties to favor the Aha1 binding, leading to the full displacement of Cdc37 from complexes. After nucleotide-induced conformational changes are established, the Hsp90-NTDs are dimerized leading to the formation of the closed intermediate state. Aha1 accelerates the ATPase cycle by facilitating dimerization process of the Hsp90-NTDs and the formation of a partially closed state with the dynamically associated ATP. Hsp90 reaches a fully closed state in which ATP hydrolysis occurs. After ATP is hydrolyzed, Aha1 is released from the complex and the Hsp90-NTDs dissociate leading to formation of the semi-open ADP-bound state. At the final state, ADP is released and Hsp90 comes back to the nucleotide-free open state. The Hsp90 structure is shown in a surface representation with a detailed annotation of structural elements. The Hsp90-NTD is shown in green; the Hsp90-MD is depicted in blue and the Hsp90-CTD is presented in red.
(TIF)

Figure S2 **Structural Characterization of the Cdc37 Chaperone.** (A) The crystal structure of human Cdc37 dimer from the complex with the Hsp90-NTDs of yeast Hsp90 (pdb id 1US7) [23]. The Cdc37 monomers are colored in cyan and green. (B) The NMR structure of the complex of the human Cdc37-MD with the N-terminal domain of human Hsp90 (pdb id 2K5B) [25]. The Hsp90-NTD (residues 14–223) is in green ribbons, the Cdc37-MD (residues 148–276) is in cyan ribbons.
(TIF)
