# Supplementary figures and images for: Allosteric Regulation of the Hsp90 Dynamics and Stability by Client Recruiter Cochaperones: Protein Structure Network Modeling
Source: PLoS One. 2014 Jan 20;9(1):e86547. doi: 10.1371/journal.pone.0086547 (PMC3896489; doi:10.1371/journal.pone.0086547)

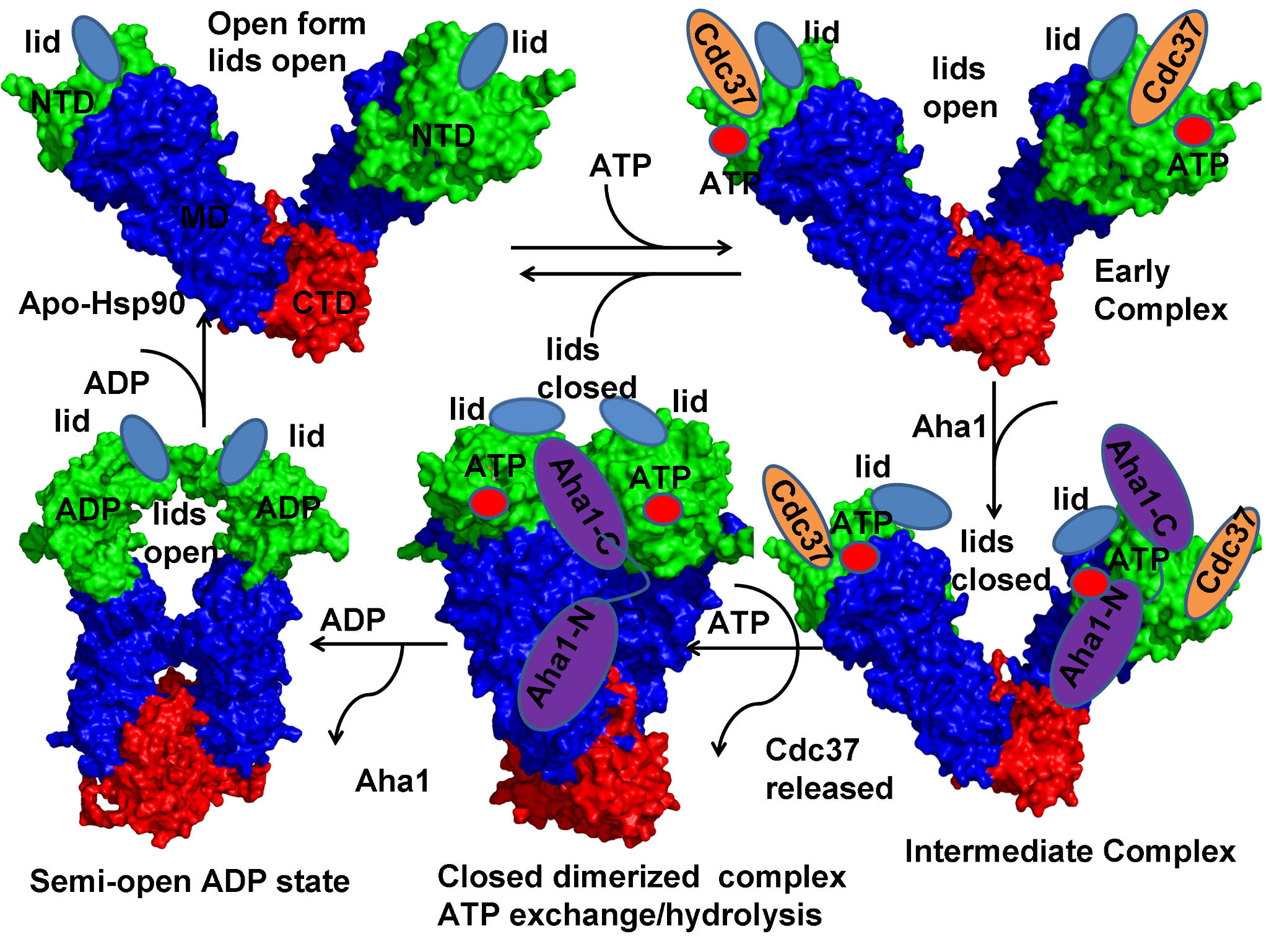

Supplement: Figure S1 — The Hsp90-Cdc37 Chaperone Cycle. Clockwise from top left, ATP binding to the Hsp90-NTD of the apo Hsp90 in the open form induces a fast dynamic exchange between a nucleotide-free Hsp90 and an intermediate ATP-bound state in which the ATP lids and Hsp90-NTDs are still open. The kinase-specific cochaperone Cdc37 acts early in the chaperone cycle and binds to the Hsp90-NTDs inducing a partially contracted open form of Hsp90 and stabilizing the nucleotide-free chaperone conformation. Protein kinase clients are recruited to the Hsp90 system though the action of Cdc37. After ATP binding, Hsp90 reaches the next intermediate state in which the ATP lids are closed but the Hsp-90-NTDs are still open. Starting from Cdc37-Hsp90 complexes, ATP binding results in an open-closed equilibrium. The hydrolysis is inhibited by Cdc37. In an intermediate ATP-bound complex, with the ATP lids closed but the Hsp90-NTDs are still separated, the cochaperone Aha1 binds to the Hsp90-MD via its N-terminal domain and begins to compete with Cdc37 binding. ATP binding shifts the binding properties to favor the Aha1 binding, leading to the full displacement of Cdc37 from complexes. After nucleotide-induced conformational changes are established, the Hsp90-NTDs are dimerized leading to the formation of the closed intermediate state. Aha1 accelerates the ATPase cycle by facilitating dimerization process of the Hsp90-NTDs and the formation of a partially closed state with the dynamically associated ATP. Hsp90 reaches a fully closed state in which ATP hydrolysis occurs. After ATP is hydrolyzed, Aha1 is released from the complex and the Hsp90-NTDs dissociate leading to formation of the semi-open ADP-bound state. At the final state, ADP is released and Hsp90 comes back to the nucleotide-free open state. The Hsp90 structure is shown in a surface representation with a detailed annotation of structural elements. The Hsp90-NTD is shown in green; the Hsp90-MD is depicted in blue and the Hsp90-CTD is presented [file pone.0086547.s001.tif]

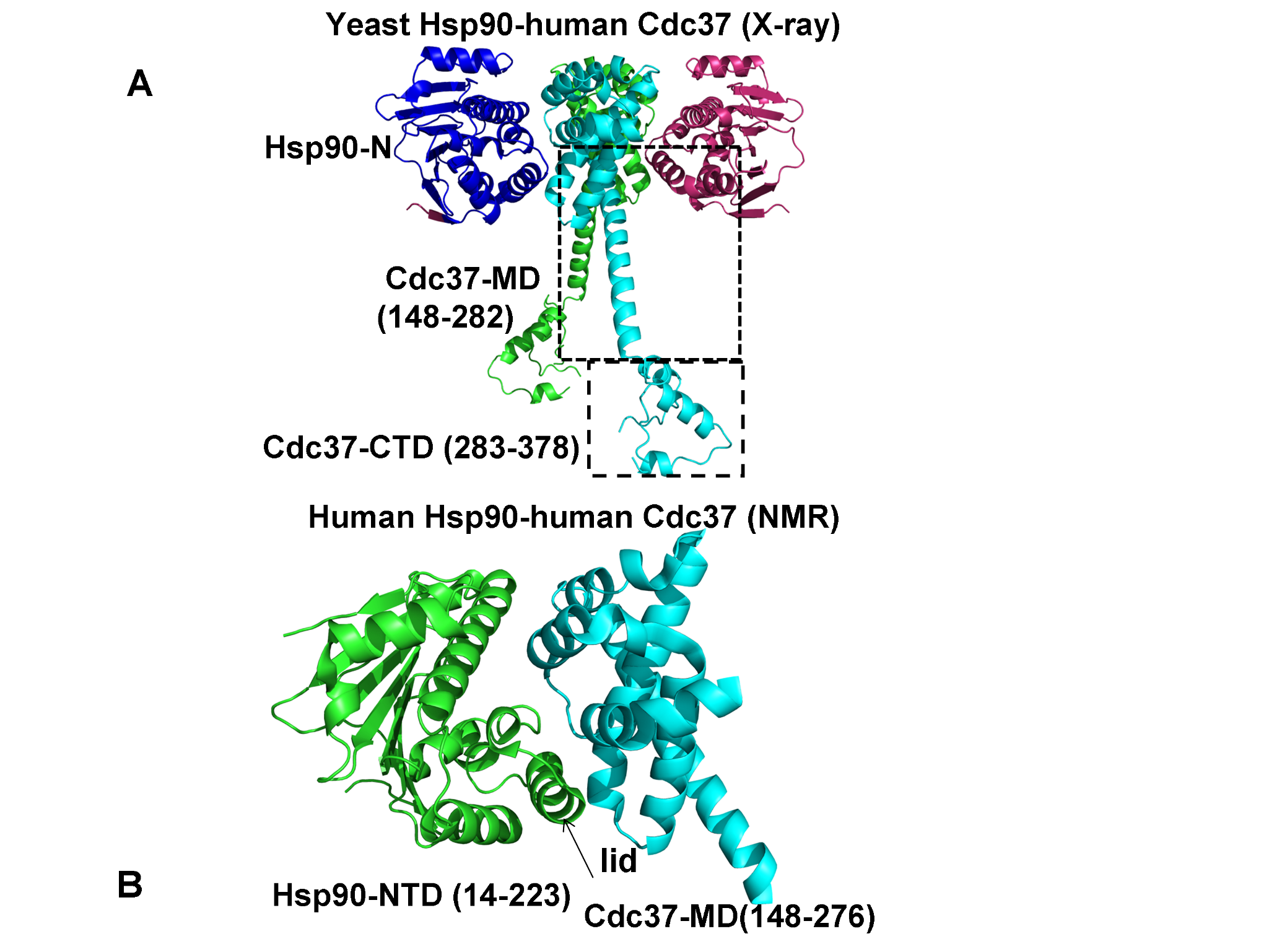

Supplement: Figure S2 — Structural Characterization of the Cdc37 Chaperone. (A) The crystal structure of human Cdc37 dimer from the complex with the Hsp90-NTDs of yeast Hsp90 (pdb id 1US7) [23]. The Cdc37 monomers are colored in cyan and green. (B) The NMR structure of the complex of the human Cdc37-MD with the N-terminal domain of human Hsp90 (pdb id 2K5B) [25]. The Hsp90-NTD (residues 14–223) is in green ribbons, the Cdc37-MD (residues 148–276) is in cyan ribbons. (TIF) [file pone.0086547.s002.tif]
